# Immune-Related lncRNAs with WGCNA Identified the Function of SNHG10 in HBV-Related Hepatocellular Carcinoma

**DOI:** 10.1155/2022/9332844

**Published:** 2022-07-06

**Authors:** Jie Hou, Zhan Wang, Hong Li, Hongzhi Zhang, Lan Luo

**Affiliations:** ^1^The People's Hospital of Baoan Shenzhen, The 8th People's Hospital of Shenzhen, Shenzhen, China; ^2^The Affiliated Baoan Hospital of Southern Medical University, Guangzhou, China

## Abstract

**Objective:**

The hepatitis B virus (HBV) infection led to hepatitis, which was one of common reasons for hepatocellular carcinoma (HCC). The immune microenvironment alteration played a crucial role in this process. The study aimed to identify immune-related long noncoding RNAs (lncRNAs) in HBV-related HCC and explore potential mechanisms.

**Methods:**

In total, 1,072 immune‐related genes (IRGs) were enriched in different co-expression modules with weighted gene co-expression network analysis (WGCNA) combining the corresponding clinical features in HBV-related HCC. The immune-related lncRNAs were selected from the crucial co-expression model based on the correlation analysis with IRGs. The immune-related lncRNAs were furtherly used to construct prognostic signature by the Cox proportional hazards regression and Lasso regression. Furthermore, the proliferation and migration ability of lncRNA SNHG10 were verified in vitro.

**Results:**

A total of nine co-expression modules were identified by WGCNA of which the “red” co-expression module was most correlated with various clinical characteristics. Additionally, the IRGs in this module were significantly enriched in multiple immune-related pathways. The twelve immune-related lncRNAs prognostic signature (HAND2-AS1, LINC00844, SNHG10, MALAT1, LINC00460, LBX2-AS1, MIR31HG, SEMA6A-AS1, LINC1278, LINC00514, CTBP-AS2, and LINC00205) was constructed. The risk score was an independent risk factor in HBV-related HCC and verified by principal components analysis (PCA), nomogram, and PCR between different cell lines. Moreover, the proportion of immune cells were significantly different between high-risk score group and low-risk score group. The malignant behavior of Hep3B was significantly different between si-lncRNA SNHG10 and control group.

**Conclusions:**

The immune-related lncRNAs prognostic signature provided some potential biomarkers and molecular mechanisms in HBV-related HCC.

## 1. Introduction

The history of human existence and development was also a “history of struggle” against viruses. Hepatitis is one of the most serious challenges that is common reason for hepatocellular carcinoma (HCC). A total of 70% of patients with HCC were detected to be infected by hepatitis B virus (HBV) and/or hepatitis C virus (HCV) [1]. Humans have figured out the “full landscape” of several hepatitis viruses, and the discovery of HBV and HCV has been awarded Nobel Prize in physiology and medicine [2]. Statistics on chronic HBV infection in 2017 showed that HBV-infected patients reached at least 391 million people of the world's population [3]. About 300,000 people die of HBV-related HCC each year, which was most common in the Western Pacific (6.2%) and African (6.1%) regions [4]. HBV was first discovered in 1966. Chronic persistent hepatitis caused by HBV can lead to liver cirrhosis, fibrosis, and cancer, which was well known as trilogy in HCC [5]; therefore, the relationship between HBV, chronic persistent hepatitis, and liver cancer needs further investigation. HBV infection can involve in HCC oncogenesis through indirect and direct mechanisms. In this process, HCC-promoting HBV factors were various, including high levels of HBV replication, HBV integration, long-lasting infection, specific HBV mutants, HBV genotype, and HBV-encoded oncoproteins (e.g., truncated preS2/S proteins and HBx). Moreover, liver inflammation can promote accumulation of mutations during host immune responses to chronic HBV infection [6]. The patients with HBV infection could activate Janus kinase (JAK) signaling pathway, STAT signaling pathway, and the Toll-like receptors pathway in hepatocytes to promote hepatocyte proliferation, inhibit hepatocyte apoptosis, and trigger liver cancer [7]. The alteration and mechanisms of tumor microenvironments in HBV-related HCC might influence the immune response to eliminate the virus or reject the tumors [8]. However, the immune response caused by HBV and its role in the mechanisms of HCC remain unclear.

The clinical benefits of immune oncology are impressive in different malignancies; however, due to the molecular and genetic heterogeneity of tumors, the application of traditional clinical and pathological criteria is far from satisfactory [9]. Weighted gene co-expression network analysis (WGCNA) is used to evaluate highly correlated gene clusters (co-expression modules) to investigate the relationship between gene co-expression modules and clinical phenotypes, based on microarray data or RNA sequencing data [10]. Besides, WGCNA is generally accepted for studying the immune-related pathways and biomarkers in HCV-related HCC. The previous study showed that SLA2547, EFNA4, and MME might play important roles in HCV-related HCC, which provided the function of gene network and a deeper understanding of HCV-related HCC [11]. The immune system was one of key factors during tumor development and progression of hepatocellular carcinoma, including alteration in the number of immune cells, dysregulation of immune-related genes, the release of chemokine and cytokine, and the dysfunction of immune cells [12]. Long noncoding RNAs (lncRNAs) are defined as a large and diverse class of RNA transcripts with a length of more than 200 nucleotides with no significant protein-coding capacity [13]. Previously published studies reported the relationship between various lncRNAs and HCC (16); that is, HULC activates HBV by modulating HBx/STAT3/miR-539/APOBEC3B signaling in HBV-related HCC [14]. Novel immune-related lncRNAs involved in the initiation and progression of HBV-related HCC need to be investigated, which would help HCC patient outcome.

In this study, we conducted a comprehensive genomic analysis to further investigate potential key co-expression modules and immune-related pathways in HBV-related HCC. The red co-expression module was correlated with some key clinical traits, such as neoplasm histologic grade, pathological tumor size (pT), pathological stage, fetoprotein value, and vascular infiltration. The IRGs in the red co-expression module were furtherly used to identify immune-related lncRNAs in HBV-related HCC. Based on the results, we suggested a signature prognostic model of twelve lncRNAs that was significantly related to the overall survival, which might help to better understand the molecular mechanisms underlying HBV-related HCC. Among those twelve lncRNAs, SNHG10 were selected to verify the function in HCC cell lines. The study was the first time to focus immune-related lncRNA in HBV-related HCC. The alteration and related mechanisms of tumor microenvironments in HBV-related HCC might influence the immune response to eliminate the virus or reject the tumors. In this study, the immune-related lncRNAs prognostic signature provided some potential biomarkers and molecular mechanisms in HBV-related HCC.

## 2. Materials and Methods

### 2.1. HBV-Related HCC Patients and Immune-Related Genes from TCGA

Human transcriptome data (including mRNAs and lncRNAs) and corresponding clinical data of 105 HBV-related HCC patients were selected and obtained from the TCGA GDC (http://cancergenome.nih.gov/). The clinical characteristics of HBV-related HCC patients included age at initial pathological diagnosis, weight, gender neoplasm histologic grade, pathological metastasis (pM), pathological node metastasis (pN), pathological tumor size (pT), pathological stage, Pugh-Child score (Class *A* = 5-6 points; Class *B* = 7–9 points; and Class *C* = 10–15 points. Class *A* means good operative risk; Class *B* means moderate operative risk; and Class *C* means poor operative risk), cancer status, adjacent hepatic tissue, inflammation extent type, albumin levels, platelet count, prothrombin time value, relative family cancer history, fetoprotein value, Ishak fibrosis stage, and vascular infiltration. A total of 1,072 immune-related genes lists were downloaded from the Immunology Database and Analysis Portal (ImmPort) (https://immport.niaid.nih.gov and Supplementary Table 1). The expression of immune-related genes from ImmPort and corresponding clinical data were used to construct the immune-related co-expression modules with WGCNA.

### 2.2. WGCNA of HBV-Related HCC and Selection of Immune-Related lncRNAs

The outlier of HBV-related HCC samples in TCGA was checked using the “flashClust” package in R 3.4.4 (http://www.r-project.org/). Some HBV-related HCC samples with greater variability were removed according to “cut height.” Dendrogram and trait-related heatmap were plotted by “WGCNA” R package (https://cran.r-project.org/web/packages/WGCNA/index.html) to explore the relationship between immune-related genes and the corresponding clinical phenotype in HBV-related HCC patients. The Pearson correlation method was used to test the correlation between the immune-related co-expression modules and eleven HBV-related HCC clinical characteristics (age at initial pathological diagnosis, weight, gender neoplasm histologic grade, pathological metastasis (pM), pathological node metastasis (pN), pathological tumor size (pT), pathological stage, Pugh-Child score, cancer status, adjacent hepatic tissue, inflammation extent type, albumin levels, platelet count, prothrombin time value, relative family cancer history, fetoprotein value, Ishak fibrosis stage, and vascular infiltration). The Kyoto Encyclopedia of Genes and Genomes (KEGG) pathway enrichment analysis was used to construct significant pathways (adjusted *p*-value <0.05) of the selected immune-related co-expression module with the DAVID 6.8 analysis tool (https://david.ncifcrf.gov/). The lncRNAs that correlated with immune-related genes of HBV-related HCC (Pearson's correlation coefficient >0.7 and *p* < 0.05) were selected using RStudio (https://www.statmethods.net/stats/correlations.html).

### 2.3. Lasso Regression for HBV-Related HCC

Immune-related lncRNAs for the overall survival (*p* < 0.05) were selected based on the Cox proportional hazards regression model. Lasso regression was constructed to test the correlation between the prognostic model of immune-related lncRNAs and HBV-related HCC risk. The best subset selection and the connections between lasso coefficient estimates were used to construct the prognostic model. Cox proportional hazards regression model (univariate and multivariate) was used to associate immune-related lncRNA signature with the overall survival in HBV-related HCC.

### 2.4. Validation of Immune-Related lncRNA Signature

The selected patients were clustered into the high-risk score group (HR) and the low-risk score group (LR). The Kaplan–Meier survival analysis method was used to evaluate the effectiveness of the prognostic model. The “Pheatmap” package in R was used to associate the expression of lncRNAs (HAND2-AS1, LINC00844, SNHG10, MALAT1, LINC00460, LBX2-AS1, MIR31HG, SEMA6A-AS1, LINC1278, LINC00514, CTBP-AS2, and LINC00205) with HR and LR. Principal component analysis (PCA) of HBV-related HCC cohort demonstrated a different distribution pattern in HR and LR based on the immune-related lncRNA expression. HR patients were denoted with red dots and LR patients with green dots. The nomogram, including risk score, age, weight, gender, neoplasm histologic grade, pathologic M, pathologic N, pathologic T, Pugh-Child classification grade, cancer status, and fetoprotein outcome value, was constructed to evaluate survival rate (1-year survival rate, 2-year survival rate, and 3-year survival rate) in HBV-related HCC patients.

### 2.5. Cell Lines and qPCR Verification

The two human hepatoblastoma cell lines and normal control cell line were used to verify the expression of the identified immune-related lncRNAs. Chronic HBV-producing HB611 cells were purchased from Keibai Academy of Science (Nanjing, China) and cultured in DMEM medium (Corning, NY, USA) plus 10% fetal bovine serum (FBS, Gibco). The hepatocellular carcinoma Hep3B cell line, which contains an integrated hepatitis B virus (HBV) genome and constitutively secretes HBsAg, was also purchased from Keibai Academy of Science (Nanjing, China) and cultured in RPMI-1640 medium (Corning, NY, USA) plus 10% fetal bovine serum (FBS, Gibco). The normal hepatocytes HL7702 were purchased from ScienCell Research Laboratory (Shanghai, China) and cultured in RPMI-1640 medium (Corning, NY, USA) plus 10% fetal bovine serum (FBS, Gibco). The total RNA of those human hepatoblastoma cell lines were extracted with TRIzol Reagent (Invitrogen) and do qRT-PCR analysis with SYBR qPCR kit (TaKaRa). All the primers (forward and reverse primers) were synthesized by Sangon Biotech (Shanghai, China).

### 2.6. Transient Transfection and Cell Test In Vitro

The Hep3B cell line was seeded in 6-well plates at 30–50% density. Transient transfection was performed with Lipofectamine 3000 reagents according to the manufacturer's instructions (Invitrogen, USA). After si-lncRNA SNHG10 and si-control treatment for 48 h, cells were used to measure DNA synthesis with a Cell-Light™ EdU imaging detecting kit (RiboBio, Guangzhou, China) according to the manufacturer's instructions. The transfected Hep3B cells were also seeded in 96-well plates according to 2000/well. After the cells were stuck to the cell walls, cells were cultured for 0 h to 48 h. Each point-in-time (0 h, 24 h, and 48 h), 10 *μ*l CCK8 was added into each well and completely shook and put into the incubator. 2 h later, 96-well plate was put into a preheated microplate reader. The absorbance values (OD) were measured at a wavelength of 450 nm in each well. Cell migration assay was assessed by using transwell chamber (Corning, NY, USA) in 24-well plate. Each group of cells (3 × 10^4^ cells/100 *μ*l) was resuspended in serum-free medium and seeded onto the upper chamber with 12.5% Matrigel-coated membrane (BD Bioscience, San Jose, CA) at 48 h post-transfection, while the lower chamber was filled with 500-*μ*l fresh complete medium. After incubated for 6–10 h at 37°C with 5% CO_2_, noninvading cells were removed from the upper surface of the filter by scraping with a cotton swab. Invading cells that adhered to the lower surface of the chambers were fixed in methyl alcohol and stained with hematoxylin. The invading cells were manually counted at 200x magnification in three random fields by using inverted microscope.

### 2.7. The Proportion of Immune Cell Infiltration between the High-Risk Score Group and the Low-Risk Score Group

To quantify the proportion of immune cell infiltration in the HBV-related HCC samples, the CIBERSORT algorithm with 22 human immune cells (B cells memory, B cells naïve, dendritic cells activated, dendritic cells resting, eosinophils, macrophage M0, macrophage M1, macrophage M2, mast cells activated, mast cells resting, monocytes, neutrophils, NK cells activated, NK cells resting, plasma cells, T cells CD4 memory activated, T cells CD4 memory resting, T cells CD4 naïve, T cells CD8, T cells follicular helper, T cells gamma delta, and T cells regulatory) was used. The expression profiles of HBV-related HCC were uploaded to the CIBERSORT web portal (http://cibersort.stanford.edu/) for 1,000 permutations. The correlation analysis between those immune cells in HBV-related HCC samples was conducted with Corrplot R package (https://cran.r-project.org/web/packages/corrplot/index.html). The different proportion of immune cells was plotted between the HR and LR.

### 2.8. Statistical Analysis

All data were processed under the WGCNA R window. When *p* < 0.05, it would be considered as statistically significance. For KEGG pathway analysis, *p* value was adjusted by Benjamini–Hochberg for multiple testing. The experiments for qPCR were totally repeated for 3 times. The differences between HBV-related cell lines and control group were analyzed by Student's *t*-test in SPSS (SPSS Inc., Chicago, USA), with statistical significance (*p* < 0.05).

## 3. Results

### 3.1. Construction of Co-Expression Modules of Immune-Related Genes in HBV-Related HCC

**Figure 1:**
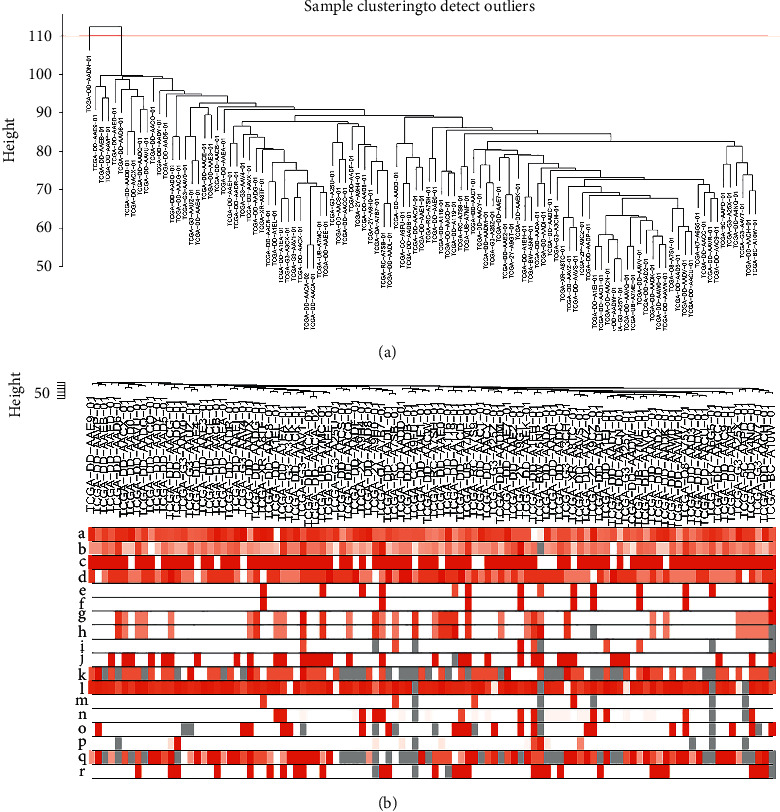
Cluster analysis of immune-related mRNAs in hepatitis B virus (HBV)-related hepatocellular carcinoma (HCC) obtained from the cancer genome atlas database. (a) Sample clustering for detecting outliers based on immune-related mRNA data. The red line represents the cutoff of data filtering in the step of data preprocessing. (b) Sample dendrogram and trait heatmap based on immune-related mRNA expression data and clinical traits. a. Age at initial pathological diagnosis, b. weight, c. gender, d. neoplasm histologic grade, e. pathological metastasis (pM), f. pathological node metastasis (pN), g. pathological tumor size (pT), h. pathological stage, i. Pugh–Child score, j. cancer status, k. adjacent hepatic tissue, l. albumin levels, m. platelet count, n. prothrombin time result value, o. relative family cancer history, p. fetoprotein value, q. Ishak fibrosis stage, and r. vascular infiltration.

**Figure 2:**
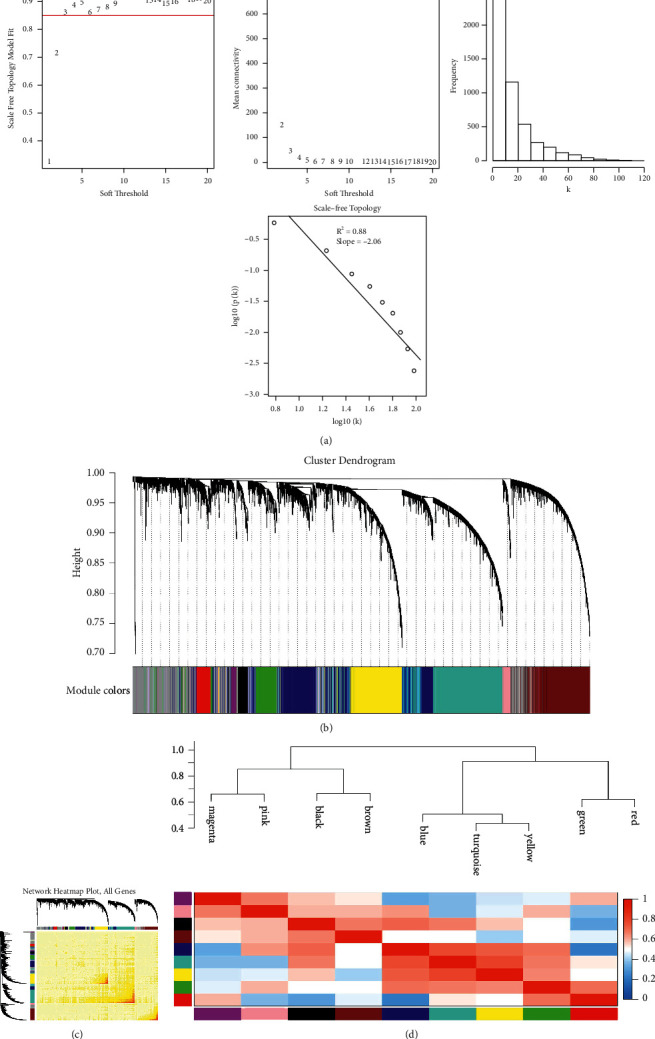
Detection of co-expression modules of hepatitis B virus (HBV)-related hepatocellular carcinoma (HCC). (a) Network topology analysis of different soft-threshold power values, including the scale-free fit index (*y*-axis) and the average connectivity degree (*y*-axis). Scale-free topology and adjacency matrix were defined by using the soft-thresholds of *β* = 3. (b) Clustering tree of different immune-related mRNAs with dissimilarity based on topological overlap along with assigned module colors. Nine co-expression modules were identified. (c) Gene topological overlap matrix heat map based on co-expression modules. (d) Heatmap of the gene network.

The expression information of immune-related genes (*n* = 1,072) in 105 HBV-related HCC samples were used for WGCNA analysis (Supplementary Table 1). Samples with missing data of more than 20% (gene expression value = 0) were removed from the analysis. Clinical characteristics of eligible HBV-related HCC patients are summarized in Supplementary Table 2. The “flashClust” R package was used to perform sample clustering, and the HBV-related HCC samples were clustered with the average linkage and Pearson's correlation method. Sample clustering was used to detect outliers (cut height = 110) based on the RNA expression data with algorithm method (Figure 1(a)). A sample dendrogram and a trait heatmap were constructed based on the sample distribution and the corresponding clinical data (age at initial pathological diagnosis, weight, gender, neoplasm histologic grade, pathological metastasis, pathological node metastasis, pathological tumor size, pathological stage, Pugh-Child score, cancer status, adjacent hepatic tissue, albumin levels, platelet count, prothrombin time result, relative family cancer history, fetoprotein value, Ishak fibrosis stage, and vascular infiltration) of HBV-related HCC patients (Figure 1(b)). Analysis of network topology for various soft-threshold powers, including the scale-free fit index (*y*-axis) and the mean connectivity (degree, *y*-axis), showed that a power value (*β*) of 3 was the optimum for the construction of distinct co-expression modules (Figure 2(a)). A cluster dendrogram of all selected genes was constructed based on a topological overlap matrix, and the co-expression modules were displayed in different colors (Figure 2(b)). The network heatmap plot of all genes and module assignment are shown in Figure 2(c). The hierarchical clustering method of module eigengenes was used to form branches in the dendrogram (meta-modules) and summarize the similarity of the modules. Each row and column in the heatmap corresponded to one module eigengene (Figure 2(d)). Overall, we identified nine distinct gene co-expression modules (magenta, pink, black, brown, blue, turquoise, yellow, green, and red) in HBV-related HCC, and each gene co-expression module contains different IRGs (Supplementary Table 3).

### 3.2. Gene Co-Expression Module Corresponds to Clinic Traits

**Figure 3:**
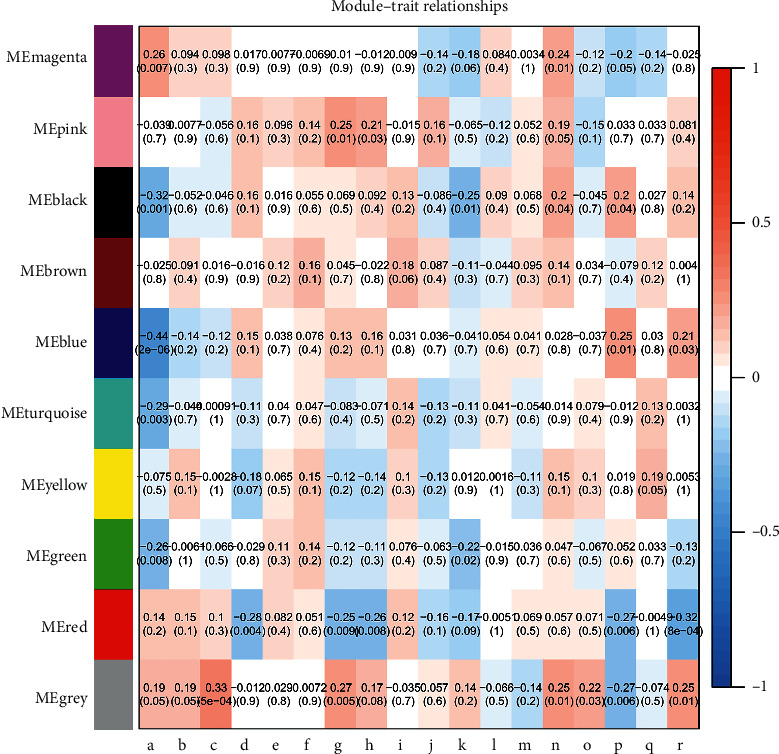
Analysis of module-trait relationships of hepatocellular carcinoma (HCC) associated with hepatitis B virus (HBV) based on the cancer genome atlas data. Each row corresponds to a module eigengene and each column to a clinical characteristic. a. Age at initial pathological diagnosis, b. weight, c. gender, d. neoplasm histologic grade, e. pathological metastasis (pM), f. pathological node metastasis (pN), g. pathological tumor size (pT), h. pathological stage, i. Pugh-Child score, j. cancer status, k. adjacent hepatic tissue, l. albumin levels, m. platelet count, n. prothrombin time value, o. relative family cancer history, p. fetoprotein value, q. Ishak fibrosis stage, and r. vascular infiltration.

A correlation analysis was conducted to evaluate possible relationships between the common expression eigengene pattern of clinical characteristics and each co-expression module. A heatmap of the correlation between module eigengenes and clinical traits is shown in Figure 3. Multiple clinical characteristics were significantly related to the red co-expression module, including histologic grade (*r* = −0.28, *p*=0.004), pathological tumor size (*r* = −0.25, *p*=0.009), pathological stage (*r* = −0.26, *p*=0.008), fetoprotein value (*r* = −0.27, *p*=0.006), and vascular infiltration (*r* = −0.32, *p*=8*e* − 04). The histologic grade included GX grade that cannot be accessed; G1 that was well differentiated; G2 that was moderately differentiated; G3 that was poorly differentiated; and G4 that was undifferentiated. The pathological tumor size included TX with primary tumor cannot be assessed; T0 with no evidence of primary tumor; T1 with solitary tumor ≤2 cm, or >2 cm without vascular invasion; T1a with solitary tumor ≤2 cm; T1b with solitary tumor >2 cm without vascular invasion; T2 with solitary tumor >2 cm with vascular invasion, or multiple tumors, none >5 cm; T3 with multiple tumors, at least one of which is >5 cm; and T4 with single tumor or multiple tumors of any size involving a major branch of the portal vein or hepatic vein, or tumor(s) with direct invasion of adjacent organs other than the gallbladder or with perforation of visceral peritoneum. The pathological stage included stage IA, stage IB, stage II, stage IIIA, stage IIIB, stage IVA, and stage IVB.

### 3.3. Pathway Enrichment Analysis of Genes in the Red Co-Expression Module

**Figure 4:**
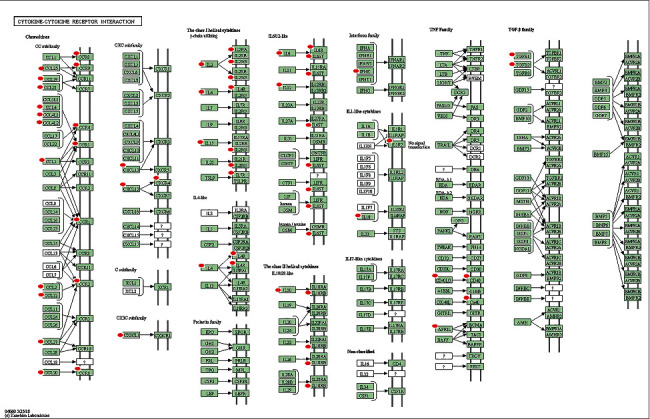
Cytokine-cytokine receptor interaction pathway altered in hepatitis B virus (HBV)-related hepatocellular carcinoma (HCC). Green rectangles with red marks depict the identified molecules.

In total, 12 significant signaling pathways (*p* < 0.05), including cytokine-cytokine receptor interaction, intestinal immune network for IgA production, chemokine signaling pathway, T-cell receptor signaling pathway, NF-kappa B signaling pathway, JAK-STAT signaling pathway, staphylococcus aureus infection, Toll-like receptor signaling pathway, TNF signaling pathway, NOD-like receptor signaling pathway, B-cell receptor signaling pathway, and RIG-I-like receptor signaling pathway, were identified in the red co-expression module (Supplementary Table 4). The highest number of immune-related genes was detected in the cytokine-cytokine receptor interaction pathway (Figure 4). Cytokines are soluble extracellular proteins or glycoproteins. They are crucial intercellular regulators and mobilizers involved in innate and adaptive inflammatory host defense, cell growth, cell differentiation, cell death, angiogenesis, and development and repair processes designed to restore homeostasis. Cytokines are released by various cells in the body, usually in response to a stimulus, which induce responses via binding to specific receptors on the surface of target cells. Therefore, further research is necessary to investigate the role of the cytokine-cytokine receptor interaction pathway in HBV-related HCC. Of course, other identified immune-related pathways were also meaningful to be further studied in HBV-related HCC.

### 3.4. The Identification of Immune-Related lncRNAs

The expression of lncRNAs in HBV-related HCC was obtained from TCGA website. A total of 178 highly correlated (|correlation coefficient| ≥ 0.7, *p* < 0.05) immune-related gene-lncRNA pairs were identified and used for further analysis (Supplementary Table 5). Based on the Cox proportional hazards regression model, 33 immune-related lncRNAs (A1BG-AS1, AC006942.1, CTBP-AS2, SNHG10, AC012146.1, HAND2-AS1, AC016044.1, AC021074.3, AC025171.1, LINC00514, MALAT1, AC083809.1, LINC00205, AC090152.1, LBX2-AS1, LINC00460, AC100847.1, AC105942.1, LINC00844, ADORA2A-AS1, AL031316.1, MIR31HG, AL133243.2, AL157373.2, SEMA6A-AS1, AL355574.1, AL360181.1, AL603839.3, AP003119.1, LINC1278, AP003469.2, C5orf56, and CARD8-AS1) were correlated (*p* < 0.05) with the overall survival (Supplementary Table 6).

### 3.5. Lasso Regression Identified the Prognostic Model of Twelve Immune-Related lncRNA Signature

**Figure 5:**
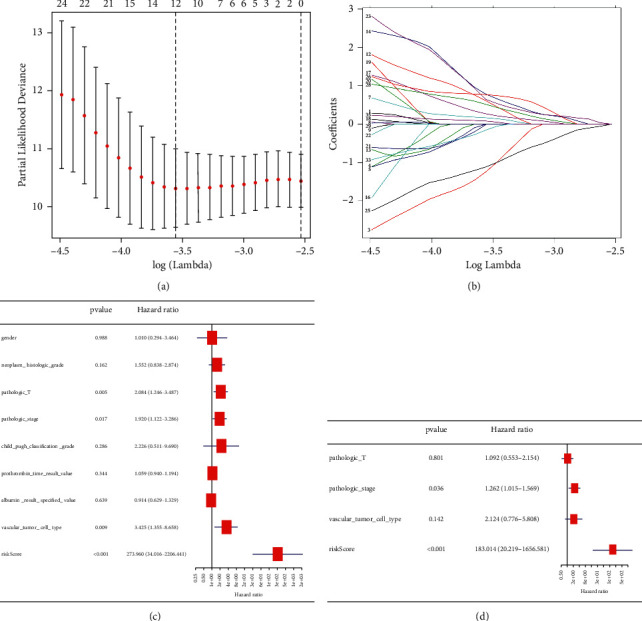
Univariate and multivariate analysis of risk factors in hepatitis B virus (HBV)-related hepatocellular carcinoma (HCC). (a, b) Lasso regression complexity controlled by lambda. (c) Univariate analysis of risk factors in HBV-related HCC. (d) Multivariate analysis of risk factors in HBV-related HCC.

Using lasso regression, we developed an optimal prognostic model of 12 immune-related lncRNAs (HAND2-AS1, LINC00844, SNHG10, MALAT1, LINC00460, LBX2-AS1, MIR31HG, SEMA6A-AS1, LINC1278, LINC00514, CTBP-AS2, and LINC00205) with log(*λ*) between −3.5 and −4 (Figures 5(a) and 5(b)) and a coefficient between −1.23 and 0.78 (Supplementary Table 6). Univariate analysis revealed that the risk score (hazard ratio, 273.96; 95% confidence interval, 34–2206; *p* < 0.001; Figure 5(c)) was correlated with poor HCC prognosis, whereas multivariate analysis revealed that the risk score was an independent prognostic marker (hazard ratio, 183.014; 95% confidence interval, 20–1656; *p* < 0.001; Figure 5(d)).

### 3.6. Twelve Immune-Related lncRNA Signature Was a Good Model

**Figure 6:**
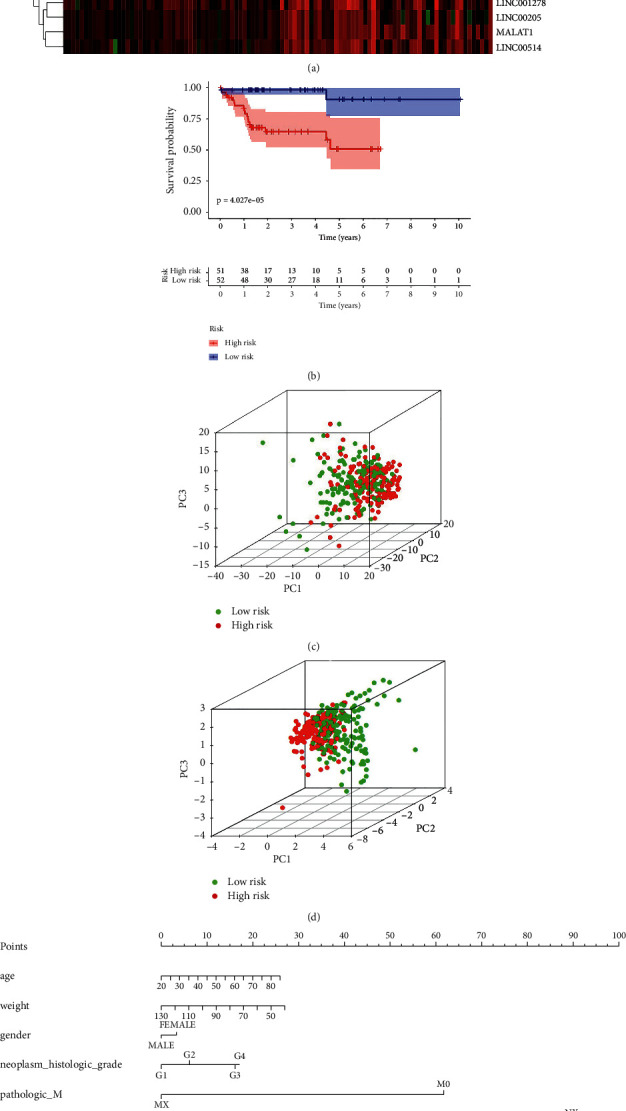
Risk score constructed in HBV-related HCC. (a) Heatmap of HBV-related HCC samples based on the risk score. Red and green points depict the risk score in the high-risk (HR) and low-risk (LR) groups, respectively. (b) Survival curve of HR vs. LR (*p* < 0.05). (c) Principal component analysis of HBV-related HCC cohort based on all identified immune-related lncRNAs. Red and blue dots depict HR and LR patients, respectively. (d) Principal component analysis of HBV-related HCC cohort based on twelve identified immune-related lncRNAs. Red and blue dots depict HR and LR patients, respectively. (e) The risk score assessment nomogram to evaluate prognosis is in HBV-related HCC (1-year survival rate, 2-year survival rate, and 3-year survival rate).

The 12 immune-related lncRNAs formed the signature exhibited distinct expression diverse expression patterns, including HAND2-AS1, LINC00844, SNHG10, MALAT1, LINC00460, LBX2-AS1, MIR31HG, SEMA6A-AS1, LINC1278, LINC00514, CTBP-AS2, and LINC00205, as shown in Figure 6(a). Afterward, the KM analysis demonstrated that patients with high-risk score were correlated with a trend toward worse survival outcomes in HBV-related HCC (*p* < 0.0001, Figure 6(b)). Principal component analysis (PCA) of HBV-related HCC cohort demonstrated a different distribution pattern of high risk and low risk based on all immune-related lncRNA expression (Figure 6(c)) or 12 immune-related lncRNA expression, indicating their difference in immune phenotype (Figure 6(d)), which indicated that distribution pattern of high risk and low risk based on 12 immune-related lncRNA expression can well-distinguished samples. Furthermore, the nomogram was made to provide a more simple and convenient method for estimating the patient survival rate according to basic clinical characteristics and risk score (Figure 6(e)).

### 3.7. RT-qPCR Confirmed the Identified Twelve Immune-Related lncRNAs

**Figure 7:**
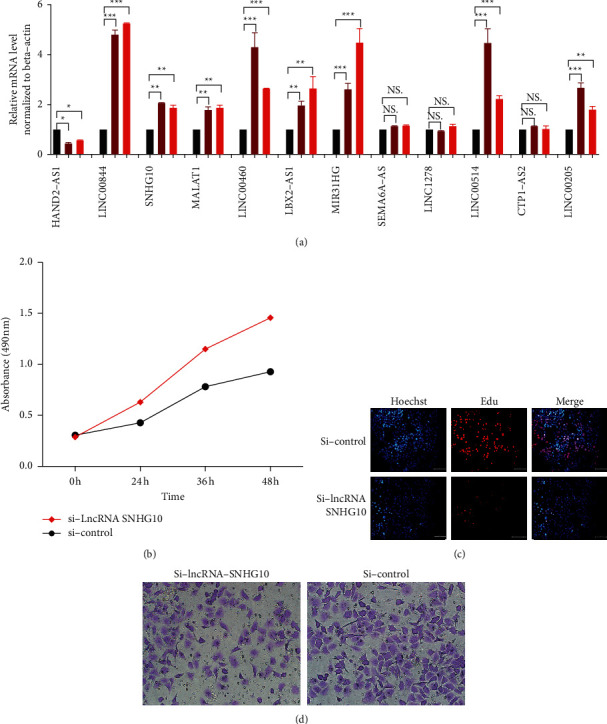
The verification of lncRNAs in prognostic model. (a) qRT-PCR analysis of 12 immune-related lncRNAs in HCC cell models compared with control cells. (b) The proliferation ability between si-lncRNA SNHG10 and control group in HepB3. (c) The DNA duplication ability between si-lncRNA SNHG10 and control group in HepB3. (d) The migration ability between si-lncRNA SNHG10 and control group in HepB3. ^*∗*^*p* < 0.05, ^*∗∗*^*p* < 0.01, and ^*∗∗∗*^*p* < 0.001.

The qRT-PCR was used to validate the expression of the identified twelve immune-related lncRNAs between human HBV-producing hepatoblastoma cell line and normal control cell line. The significant difference was found for nine immune-related lncRNAs, including eight upregulated immune-related lncRNAs (LINC00844, SNHG10, MALAT1, LINC00460, LBX2-AS1, MIR31HG, LINC00514, and LINC00205) and one downregulated immune-related lncRNA (HAND2-AS1) between human HBV-producing hepatoblastoma cell lines (chronic HBV-producing HB611 and Hep3B) and normal control cell line (HL7702), as shown in Figure 7(a).

### 3.8. The Proliferation and Migration Ability of lncRNA SNHG10 In Vitro

To evaluate the biological functions of SNHG10 in the development of HBV-related HCC, we conducted loss-of-function studies in Hep3B cells by transient transfection with si-lncRNA SNHG10 and control. Low expression of SNHG10 in HCC cell lines remarkably decreased the rate of proliferation with CCK8 (Figure 7(b)) assay and DNA replication with EdU assay (Figure 7(c)), and enhanced the number of migratory cells with Transwell assay (Figure 7(d)).

### 3.9. The Distribution of Immune Cells in Different Risk Score Subtypes

**Figure 8:**
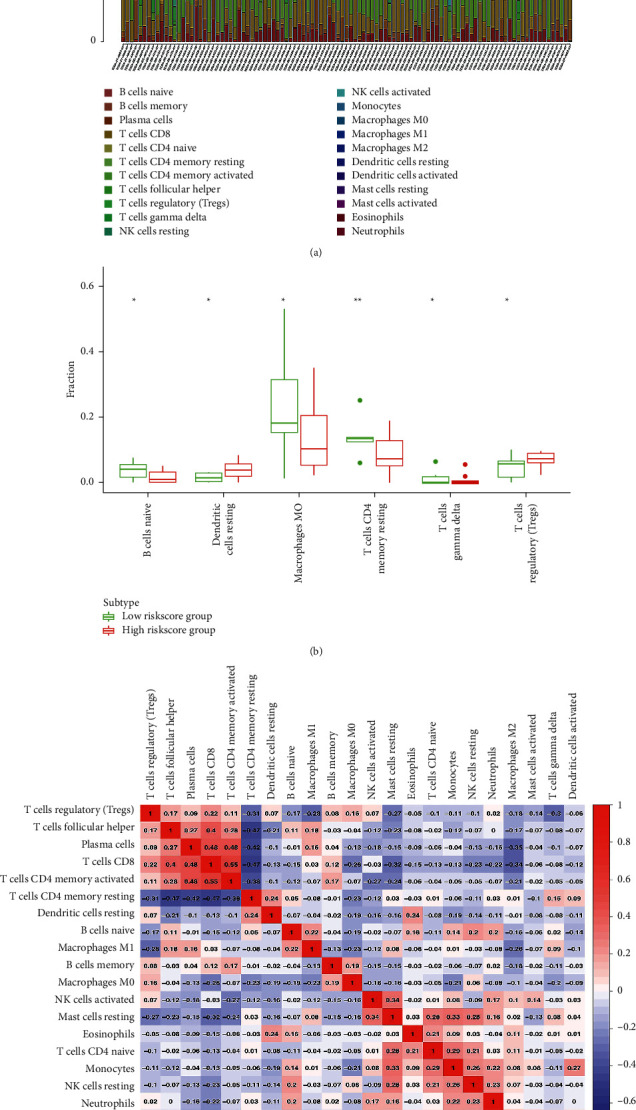
The distribution and correlation of immune cells in HBV-related HCC. (a) Bar plot showing the proportion of 22 kinds of immune cells in HBV-related HCC samples. Column names of plot were sample ID. (b) Boxplot showed the ratio differentiation of 6 kinds of immune cells between high- and low-risk score groups, and Wilcoxon rank sum was used for the significance test. The sample group was determined by the comparison with the median of risk score. *p* value was verified by log-rank test. ^*∗*^*p* < 0.05, ^*∗∗*^*p* < 0.01, and ^*∗∗∗*^*p* < 0.001. (c) Heatmap showing the correlation between 22 kinds of immune cells in HBV-related HCC samples and numeric in each tiny box indicating the correlation coefficients between two kinds of cells.

The tumor-immune infiltration of the 106 HBV-related HCC (Figure 8(a) and Supplementary Table 7) samples was summarized. The distribution of immune cells was significant between low-risk score group and high-risk score group in HBV-related HCC, including B cells naive, dendritic cells resting, macrophages M0, T cells CD4 memory resting, T cells gamma delta, and T cells regulatory (Figure 8(b)). The correlation analysis between immune cells was conducted with Corrplot R, and results showed some strong tumor-immune cell interactions in HBV-related HCC; for example, T cells CD4 memory resting and plasma, T cells CD4 memory resting and CD8 T cell, T cells follicular helper and T cells CD4 memory resting, macrophages M2, and mast cells activated (Figure 8(c)).

## 4. Discussion

Hepatocellular carcinoma (HCC) is a primary malignant tumor of the liver, occurring mainly in patients with chronic liver disease and cirrhosis [15]. The incidence of HCC is high in Asia and Africa because of the high prevalence of HBV and HCV infections that lead to the development of chronic liver inflammation [16]. In 1981, Beasley first linked HCC development to liver disease caused by HBV; however, the pathophysiology of HBV-related HCC is still unclear. HBV is a DNA virus that integrates into the host genome and generates HBV X protein, which may play a key regulatory role in the development of HCC [17]. HCC development is closely related to liver inflammation, necrosis, fibrosis, and cirrhosis [18]. The different regulation of HBV-associated immune responses may be related to defense mechanisms of HCC cells against the immune system. Thus, it is necessary to identify immune-related genes related to the clinical outcome of HBV-related HCC.

HBV infection is internationally recognized as a contributing factor to the initiation and progression in liver cancer [19]. The trilogy of liver cancer (hepatitis, liver cirrhosis, and hepatocarcinoma) promoted researchers to study the initiative factor, such as immune-related genes and pathway alterations in HBV-related HCC. Previous studies have shown that the HBV-encoded HBx and SATB1 may have a crucial role in promoting anoikis resistance and metastasis in HBV-associated liver cancer [20]. The enhancement of local inflammatory responses contributes to the recruitment of neutrophils and release of IL-1 beta, leading to chronic liver injury, persistence of viral infection, and the possible initiation and promotion of HCC [21]. The different modulation of immune response B virus may be related to defense mechanisms adopted by HCC cells against the immune system. It is meaningful to explore immune-related genes corresponding clinical outcome in HBV-related hepatocellular carcinoma. Some important researches have been reported. For example, HLA-DPB1 is part of the HLA class II beta chain paralogues, which are expressed in antigen-presenting cells (B lymphocytes, dendritic cells, and macrophages), and plays a key role in the immune system by presenting peptides derived from extracellular proteins [22]. HLA-DP polymorphisms affect the clearance rate of type B HBV, regulate the immune selection of viral mutations, influence cirrhosis, and increase the risk of HCC leading to HBV mutations [23]. Cytokines are a family of secreted proteins engaged in immune regulation and inflammatory processes. CCL22 may play a role in the trafficking of activated T lymphocytes to inflammatory sites and other aspects of T lymphocyte physiology [24]. Previous studies strongly suggested that HBV infection and the activity of the TGF-*β*-miR-34a-CCL22 axis are important causes for the development of portal vein thrombosis in HCC patients, possibly by creating an immune-subversive microenvironment that favor the colonization of disseminated HCC cells in the portal venous system [25]. Immune checkpoint proteins PD-1 are important for the T-cell function and conducive to the precaution of autoimmune diseases. The expression and polymorphisms of PD-1, T-cell immunoglobulin domain, and mucin domain-containing TIM-3 are known to be associated with liver disease caused by HBV and HCC. Previously published findings supported the potential to improve the efficiency of immune checkpoint-targeted therapy by blocking both PD-1 and TIM-3 [26]. As a member of the immunoglobulin superfamily, CTLA-4 encodes a protein that transmits an inhibitory signal to T cells and is involved in the immune dysfunction of liver disease caused by HBV and HCC [27]. A previous study showed that the circulating CTLA-4 levels and the CTLA4 rs231775 polymorphism are associated with the condition and progression of chronic liver disease caused by HBV and HCC, and thus, their determination may be used for prognosis and monitoring disease progression [28]. In the present study, many immune-related genes and significant signaling pathways (*p* < 0.05) were identified in the “red” co-expression module, which were closely related to the immune system and HBV-related HCC. For example, IL-17 and its epigenetic regulation (promoter methylation) during the progression of chronic hepatitis caused by HBV were proved to be associated with the progression of liver disease in HCC patients. Thus, the IL-17 promoter status may be used as a biomarker for identifying the correct treatment strategy based on the chronic hepatitis B stage [29]. The polymorphism of IL-2 gene may contribute to individual's susceptibility to HBV-related HCC trough altering cytokine production and/or activity. Additionally, the previous study showed that genotypes carrying the IL-2^+^114T/G variant allele might led to increased HBV-related HCC risk through decreasing the serum IL-2 levels [30]. The activity of TGF-*β*-miR-34a-CCL22 axis was also reported to induce venous metastases of HBV-positive HCC. Mechanistically, the upregulated TGF-*β* activity could increase the production of chemokine CCL22 by suppressing microRNA-34a, which enhanced the process of regulatory T cells to facilitate immune escape [25]. The investigation of the dynamic expression of immune-related genes at the stages of chronic liver disease, liver cirrhosis, and HCC may help to understand the relationship between viral infection, tumor microenvironment, and tumor progression.

LncRNA biomarkers are commonly identified in patients with HBV-associated HCC and are used as prognostic tools [31]. For example, the underlying mechanism of SNHG20 in HBV-related HCC showed that HBx promoted the proliferation of HCC cells and reduced their apoptosis through the SNHG20/PTEN signaling pathway Therefore, it is necessary to determine the function and mechanism of lncRNA in HBV-related HCC to identify effective prognostic biomarkers and therapeutic targets. In the present study, nine immune-related co-expression modules were identified analyzing data from 105 HBV-related HCC patients using WGCNA. Compared with other analysis methods, WGCNA has many distinct advantages since it focuses on the association between the co-expression modules and significant clinical traits [32]. Our analysis results were consistent with those reported in previous studies; for instance, lncRNA MIR31HG inhibits HCC proliferation and metastasis by sponging microRNA-575 to modulate ST7L expression [33], and HBV X protein promotes HCC by altering lncRNA expression profiles. However, we also reported some new findings. For instance, SEMA6A-AS1 expression in HBV-related HCC tissues was significantly downregulated compared with that in paracancerous tissues. Furthermore, low levels of SEMA6A-AS1 were associated with high *α*-fetoprotein levels, high stage of lymph node metastasis, and poor clinical response [34]. Both the Kaplan–Meier survival analysis method and the univariate Cox proportional hazards regression model showed that low expression of SEMA6A-AS1 was correlated with an overall poor survival rate. Importantly, lncRNA SNHG10 were furtherly verified in vitro test and were significantly influence proliferation and migration in HBV-related HCC. The mechanism of SNHG10-driven HCC cell malignant phenotype had been reported in previous study, and they suggested that SNHG10 can regulate SOX9 to influence proliferation, and facilitated the cell cycle, invasion and migration, and epithelial-mesenchymal transition in the process of hepatocarcinogenesis.

In conclusion, our study used WGCNA to link HBV, immune microenvironment, and HCC to explore potential biomarker of the immune-related lncRNAs in HBV-related HCC. We identified the red co-expression module that was correlated with five clinical traits (histology grade, pT, pathological stage, fetoprotein value, and vascular infiltration) and various immune system pathways. The function of SNHG10 was significant and may be developed as a new therapeutic target in HBV-related HCC. Additionally, the construction of immune-related lncRNAs in prognostic model may help to better understand the molecular mechanisms underlying HBV-related HCC.

## Data Availability

Publicly available datasets were used in this study. These data can be found in the gene expression omnibus (GEO) database and in the cancer genome atlas (TCGA) database.

## Supplementary Materials

Supplementary Materials Supplementary table 1: immune‐related gene expressions in HBV-related hepatocellular carcinoma from TCGA database for the WGCNA analysis. Supplementary table 2: the clinical characteristics of these eligible patients. Supplementary table 3: list of immune-related genes in the co-expression modules. Supplementary table 4: pathway analysis mapped the identification in the red co-expression module. Supplementary table 5: the co-expression analysis between immune-related genes in the red co-expression module and lncRNAs. Supplementary table 6: 33 immune-related lncRNAs were significant related to the overall survival. Supplementary table 7: lasso regression was constructed examining the relationship between gene signature and HCC risk. Supplementary table 8: quantification of the abundance of immune cell infiltration in tumor microenvironment by CIBERSORT web portal with the LM22 signature.
