# Supplementary material for: Immune-Related lncRNAs with WGCNA Identified the Function of SNHG10 in HBV-Related Hepatocellular Carcinoma
Source: J Oncol. 2022 Jul 6;2022:9332844. doi: 10.1155/2022/9332844 (PMC9279027; doi:10.1155/2022/9332844)
Supplement: Supplementary Materials — Supplementary table 1: immune‐related gene expressions in HBV-related hepatocellular carcinoma from TCGA database for the WGCNA analysis. Supplementary table 2: the clinical characteristics of these eligible patients. Supplementary table 3: list of immune-related genes in the co-expression modules. Supplementary table 4: pathway analysis mapped the identification in the red co-expression module. Supplementary table 5: the co-expression analysis between immune-related genes in the red co-expression module and lncRNAs. Supplementary table 6: 33 immune-related lncRNAs were significant related to the overall survival. Supplementary table 7: lasso regression was constructed examining the relationship between gene signature and HCC risk. Supplementary table 8: quantification of the abundance of immune cell infiltration in tumor microenvironment by CIBERSORT web portal with the LM22 signature. [file 9332844.f1.zip › Supplementary table 2.pdf]

Supplementary table 2: The clinical characteristics of these eligible pa

sampleID	age_at_initial_pathologic_diagnosis	weight	gender
TCGA-DD-AACX-01	66	53	MALE
TCGA-DD-AAD5-01	54	70	MALE
TCGA-DD-AADG-01	70	61	MALE
TCGA-DD-AADR-01	58	75	MALE
TCGA-DD-AAED-01	51	69	MALE
TCGA-UB-A7MC-01	59	78	MALE
TCGA-2Y-A9H4-01	68	61	MALE
TCGA-DD-AACW-01	43	65	MALE
TCGA-DD-AAD3-01	43	77	MALE
TCGA-DD-AADL-01	58	53	MALE
TCGA-DD-AADM-01	58	55	MALE
TCGA-DD-AADN-01	59	62	MALE
TCGA-DD-AADO-01	55	58	MALE
TCGA-DD-AADQ-01	59	64	MALE
TCGA-DD-AADV-01	50	64	MALE
TCGA-DD-AAE3-01	50	73	MALE
TCGA-DD-AAE7-01	72	74	MALE
TCGA-DD-AAE9-01	69	63	MALE
TCGA-DD-AAEA-01	65	66	MALE
TCGA-DD-AAEB-01	60	75	MALE
TCGA-2Y-A9GT-01	51	122	MALE
TCGA-2Y-A9HO-01	49	89	MALE
TCGA-O8-A75V-01	54	92	MALE
TCGA-K7-A6G5-01	66	80	MALE
TCGA-DD-A116-01	68	73	MALE
TCGA-DD-A1EA-01	68	94	MALE
TCGA-DD-A1EH-01	23	70	MALE
TCGA-DD-A1EL-01	23	54	MALE
TCGA-DD-AACA-01	65	70	MALE
TCGA-DD-AACE-01	62	62	MALE
TCGA-DD-AACN-01	32	70	MALE
TCGA-DD-AACO-01	40	43	MALE
TCGA-DD-AACQ-01	50	64	MALE
TCGA-DD-AACY-01	61	62	MALE
TCGA-DD-AAD0-01	73	54	FEMALE
TCGA-DD-AAD6-01	66	93	MALE
TCGA-DD-AADW-01	48	49	MALE
TCGA-DD-AAE4-01	49	62	FEMALE
TCGA-DD-AAEE-01	55	58	MALE
TCGA-DD-AAEI-01	72	79	MALE
TCGA-DD-AAVX-01	38	65	MALE
TCGA-G3-A25Y-01	52	57	FEMALE
TCGA-G3-A3CH-01	53	74	MALE
TCGA-G3-AAV1-01	51	99	MALE
TCGA-QA-A7B7-01	48	108	MALE
TCGA-XR-A8TF-01	74	57	MALE
TCGA-DD-AAVP-01	48	62	MALE
TCGA-DD-AAVQ-01	38	72	MALE
TCGA-DD-AAVU-01	46	88	MALE
TCGA-BC-A10W-01	50	71	MALE
TCGA-BW-A5NP-01	26		FEMALE
TCGA-CC-A9FU-01	52	48	FEMALE

TCGA-DD-A119-01	40	58	MALE
TCGA-DD-A1EI-01	46	69	MALE
TCGA-DD-AAC8-01	72	57	MALE
TCGA-DD-AAC9-01	51	81	MALE
TCGA-DD-AACB-01	74	72	FEMALE
TCGA-DD-AACC-01	61	61	MALE
TCGA-DD-AACD-01	48	66	MALE
TCGA-DD-AACG-01	52	65	MALE
TCGA-DD-AACH-01	69	53	MALE
TCGA-DD-AACK-01	70	68	MALE
TCGA-DD-AACS-01	39	80	MALE
TCGA-DD-AACT-01	69	62	FEMALE
TCGA-DD-AACU-01	59	67	MALE
TCGA-DD-AAD2-01	66	52	MALE
TCGA-DD-AADA-01	66	59	FEMALE
TCGA-DD-AADB-01	51	68	MALE
TCGA-DD-AADC-01	53	81	MALE
TCGA-DD-AADD-01	51	68	MALE
TCGA-DD-AADF-01	64	55	FEMALE
TCGA-DD-AADI-01	43	64	FEMALE
TCGA-DD-AADK-01	68	51	FEMALE
TCGA-DD-AADP-01	45	67	MALE
TCGA-DD-AADY-01	55	55	FEMALE
TCGA-DD-AAE0-01	45	56	FEMALE
TCGA-DD-AAE1-01	52	62	MALE
TCGA-DD-AAE2-01	51	82	MALE
TCGA-DD-AAEK-01	51	64	MALE
TCGA-DD-AAVR-01	44	69	MALE
TCGA-DD-AAVS-01	56	72	MALE
TCGA-DD-AAVV-01	56	71	MALE
TCGA-DD-AAVW-01	35	62	MALE
TCGA-DD-AAVZ-01	38	62	MALE
TCGA-DD-AAW0-01	54	75	MALE
TCGA-G3-A25U-01	63	47	FEMALE
TCGA-G3-A25X-01	73	65	MALE
TCGA-G3-A25Z-01	58	69	MALE
TCGA-G3-A3CK-01	61	67	MALE
TCGA-G3-AAV0-01	58	58	MALE
TCGA-G3-AAV4-01	83	56	FEMALE
TCGA-G3-AAV7-01	38	58	MALE
TCGA-RC-A7S9-01	47	58	FEMALE
TCGA-RC-A7SB-01	53	61	MALE
TCGA-RC-A7SH-01	42	68	MALE
TCGA-UB-A7ME-01	51	75	MALE
TCGA-ZP-A9CZ-01	72	73	MALE
TCGA-5C-AAPD-01	61	69	MALE
TCGA-XR-A8TC-01	43	52	FEMALE
TCGA-DD-AACA-02	65	70	MALE
TCGA-DD-AAD1-01	51	44	FEMALE
TCGA-DD-AAD8-01	73	57	FEMALE
TCGA-UB-A7MF-01	56	95	MALE
TCGA-G3-AAUZ-01	48	77	MALE
TCGA-DD-A4NQ-01	60	75	MALE

tients.

neoplasm_histologic_grade	pathologic_M	pathologic_N
G3	M0	N0
G3	M0	N0
G3	M0	N0
G3	M0	N0
G3	M0	N0
G3	MX	N0
G2	MX	N0
G3	M0	N0
G2	M0	N0
G4	M0	N0
G3	M0	N0
G4	MX	NX
G3	M0	N0
G3	M0	N0
G3	M0	N0
G2	M0	N0
G2	M0	N0
G3	M0	N0
G3	M0	N0
G2	M0	N0
G2	MX	NX
G1	M0	N0
G2	MX	NX
G2	MX	N0
G3	M0	N0
G2	M0	N0
G3	M0	N0
G3	M0	N0
G3	M0	N0
G3	M0	N0
G3	M0	N0
G3	M0	N0
G3	M0	N0
G3	M0	N0
G2	M0	N0
G3	M0	N0
G3	M0	N0
G1	M0	N0
G4	M0	N0
G2	M0	N0
G2	M0	N0
G3	M0	N0
G2	M0	N0
G3	M0	N0
G2	MX	NX
G1	MX	NX
G1	M0	N0
G2	M0	N0
G2	M0	N0
G3	MX	NX
G3	M1	N0
G2	M0	N0

G3	M1	N0
G2	M0	N0
G3	M0	N0
G2	M0	N0
G3	M0	N0
G2	M0	N0
G4	M0	N0
G4	M0	N0
G3	M0	N0
G2	M0	N0
G3	M0	N0
G2	M0	N0
G3	M0	N0
G2	M0	N0
G3	M0	N0
G4	M0	N0
G3	M0	N0
G4	M0	N0
G4	M0	N0
G3	M0	N0
G3	M0	N0
G3	M0	N0
G2	M0	N0
G4	M0	N0
G3	M0	N0
G3	M0	N0
G3	M0	N0
G2	M0	N0
G2	M0	N0
G3	M0	N0
G2	M0	N0
G2	M0	N0
G2	M0	N0
G3	M0	N0
G3	M0	N0
G2	M0	N0
G2	M0	N0
G2	M0	N0
G1	M0	N0
G2	M0	N0
G3	M0	N0
G2	M0	N0
G3	M0	N0
G2	MX	NX
G1	MX	NX
G1	M0	N0
G2	MX	NX
G3	M0	N0
G4	M0	N0
G2	M0	N0
G2	MX	NX
G2	M0	N0
G3	M0	N0

pathologic_T	pathologic_stage	child_pugh_classification_grade
T2	Stage II	A
T1	Stage I	A
T3a	Stage IIIA	A
T1	Stage I	A
T1	Stage I	A
T3a	Stage IIIA	A
T1	Stage I	A
T1	Stage I	A
T1	Stage I	A
T1	Stage I	A
T2	Stage II	A
T1	Stage I	A
T1	Stage I	A
T2	Stage II	A
T1	Stage I	A
T1	Stage I	A
T1	Stage I	A
T1	Stage I	A
T1	Stage I	A
T1	Stage I	A
T1	Stage I	C
T3	Stage IIIA	
T1	Stage I	A
T1	Stage I	
T3	Stage IIIA	A
T2	Stage II	A
T3	Stage III	B
T2	Stage II	A
T1	Stage I	A
T1	Stage I	A
T1	Stage I	A
T1	Stage I	A
T2	Stage II	A
T1	Stage I	A
T1	Stage I	A
T3a	Stage IIIA	A
T1	Stage I	A
T1	Stage I	A
T1	Stage I	A
T1	Stage I	A
T2	Stage II	A
T1	Stage I	A
T3a	Stage IIIA	A
T4	Stage IIIC	B
T2	Stage II	A
T1	Stage I	A
T1	Stage I	A
T1	Stage I	A
T2	Stage II	A
T4		
T2	Stage IV	
T3a	Stage IIIA	

T3a	Stage IV	A
T1	Stage I	B
T1	Stage I	A
T1	Stage I	A
T1	Stage I	A
T1	Stage I	A
T1	Stage I	A
T2	Stage II	A
T2	Stage II	A
T1	Stage I	A
T1	Stage I	A
T1	Stage I	A
T1	Stage I	A
T1	Stage I	A
T1	Stage I	A
T1	Stage I	A
T1	Stage I	A
T1	Stage I	A
T1	Stage I	A
T1	Stage I	A
T2	Stage II	A
T1	Stage I	A
T1	Stage I	A
T3a	Stage IIIA	A
T1	Stage I	A
T1	Stage I	A
T2	Stage II	A
T1	Stage I	A
T1	Stage I	A
T2	Stage II	A
T1	Stage I	A
T1	Stage I	A
T1	Stage I	A
T1	Stage I	A
T2	Stage II	A
T1	Stage I	A
T1	Stage I	A
T1	Stage I	A
T1	Stage I	A
T2	Stage II	A
T1	Stage I	A
T2	Stage II	B
T2	Stage II	A
T1	Stage I	B
T1		A
T2	Stage II	A
T1	Stage I	A
T1	Stage I	A
T1	Stage I	A
T1	Stage I	A
T3a	Stage IIIA	A
T1	Stage I	A
T2	Stage II	A

cancer_status	adjacent_hepatic_tissue_inflammation_extent_type
WITH TUMOR	Mild
WITH TUMOR	Mild
TUMOR FREE	Mild
TUMOR FREE	Mild
WITH TUMOR	Mild
WITH TUMOR	Mild
TUMOR FREE	
TUMOR FREE	
TUMOR FREE	Mild
TUMOR FREE	
TUMOR FREE	
TUMOR FREE	
TUMOR FREE	
TUMOR FREE	
TUMOR FREE	None
TUMOR FREE	None
TUMOR FREE	Severe
TUMOR FREE	Mild
TUMOR FREE	
TUMOR FREE	
WITH TUMOR	None
TUMOR FREE	None
TUMOR FREE	Mild
TUMOR FREE	
TUMOR FREE	
WITH TUMOR	Mild
WITH TUMOR	None
WITH TUMOR	Mild
WITH TUMOR	Mild
TUMOR FREE	Mild
WITH TUMOR	
WITH TUMOR	Mild
WITH TUMOR	
WITH TUMOR	Mild
WITH TUMOR	Mild
TUMOR FREE	
TUMOR FREE	None
WITH TUMOR	None
WITH TUMOR	None
WITH TUMOR	None
WITH TUMOR	Severe
WITH TUMOR	Mild
WITH TUMOR	Severe
WITH TUMOR	Severe
WITH TUMOR	Mild
TUMOR FREE	None
TUMOR FREE	Severe
TUMOR FREE	Severe
TUMOR FREE	Mild
WITH TUMOR	
WITH TUMOR	Mild
TUMOR FREE	

	None
TUMOR FREE	Mild
TUMOR FREE	Mild
TUMOR FREE	Mild
TUMOR FREE	Mild
WITH TUMOR	Mild
WITH TUMOR	Mild
WITH TUMOR	Mild
TUMOR FREE	Mild
TUMOR FREE	None
TUMOR FREE	
TUMOR FREE	Mild
TUMOR FREE	
TUMOR FREE	Mild
TUMOR FREE	
TUMOR FREE	
WITH TUMOR	
TUMOR FREE	
TUMOR FREE	
TUMOR FREE	
TUMOR FREE	None
TUMOR FREE	
TUMOR FREE	None
TUMOR FREE	Mild
TUMOR FREE	Mild
TUMOR FREE	None
TUMOR FREE	None
TUMOR FREE	None
TUMOR FREE	Mild
TUMOR FREE	Mild
TUMOR FREE	None
TUMOR FREE	None
TUMOR FREE	Mild
TUMOR FREE	Mild
TUMOR FREE	Mild
TUMOR FREE	Mild
TUMOR FREE	Mild
TUMOR FREE	None
TUMOR FREE	
TUMOR FREE	Mild
TUMOR FREE	Mild
TUMOR FREE	None
WITH TUMOR	None
TUMOR FREE	Mild
TUMOR FREE	
TUMOR FREE	None
WITH TUMOR	Mild
WITH TUMOR	Mild
WITH TUMOR	
TUMOR FREE	Mild
WITH TUMOR	
TUMOR FREE	Severe
WITH TUMOR	Mild

platelet_result_count	prothrombin_time_result_value	albumin_result_specified_value
179	0.9	4.2
349	1.2	3.7
170	1	4.2
75	1.1	4.4
211	1.1	4
200	0.9	3.9
223	11	4.6
137	1.1	4
181	1	3.9
204	0.9	3.5
126	1	4.1
176	1.1	3.5
180	1	4.5
163	1.1	4.4
187	1	3.8
180	1.1	4.7
188	1	4.7
138	1.1	4.1
255	1	4.5
270	1	4.1
187	9.8	3.7
207	12.2	2
211	9.1	3.8
157	1	4.1
209	10.2	3.3
212	10.4	4.7
153	10.8	4.4
136	0.9	3.7
175	0.9	4.4
185	1	3.9
88	1	3.7
157	1	4.2
114	1.1	3.1
86	1	3.4
185	1	2.7
102	1	4.3
113	1.1	4
221	1	4.7
161	1.1	3.6
186	1	3.9
166	1.1	0.4
142	1.2	0.3
115	1.2	3.2
218	11.5	4.2
194000	1.1	3.9
189	1	4.3
128	1.2	3
166	1	4.6
401	0.9	2.8

572	1	3.9
148	10.9	5.1
177	0.9	4.3
149	0.9	4.2
179	0.8	4.1
208	1	3.9
268	6.9	3.9
196	0.9	3.6
290	0.9	4.3
98	0.9	4.6
223	1	4.3
213	1	4.4
166	0.9	4.4
172	1	3.5
173	1.1	4.1
180	1	4.5
212	1.2	3.8
299	1.1	4.1
174	1.2	3.5
141	1.1	3.8
197	1	4.4
102	1.1	3.7
159	1.3	3.5
226	0.9	4.8
102	0.9	4.1
238	1	4.4
202	1	4.1
174	1	3.6
281	1.1	3
244	1.1	4.6
212	1	4.6
98	1.2	3.8
183	1	4.6
113	1	0.3
102	1	0.4
115	1	0.4
143	1	0.2
193	0.9	4.2
79	1.2	2.7
179	1	4
178000	1.1	4.3
196000	1	4.8
225	1	4.1
181	1	4.2
	11.5	4.4
318000	1	3.9
136	0.9	3.7
157	0.9	4.5
130	1.1	4.3
130	1	3.6
214	1	3.8
283	10.1	4.4

fetoprotein_outcome_value	fibrosis_ishak_score	relative_family_cancer_history
3	3,4 - Fibrous Speta	NO
73	3,4 - Fibrous Speta	NO
2256	3,4 - Fibrous Speta	NO
3	6 - Established Cirrhosis	NO
2405	1,2 - Portal Fibrosis	NO
126	1,2 - Portal Fibrosis	NO
11		YES
1726		NO
	1,2 - Portal Fibrosis	NO
14		YES
112		NO
151367		NO
176037		NO
7		NO
11	6 - Established Cirrhosis	NO
2	1,2 - Portal Fibrosis	NO
1	3,4 - Fibrous Speta	NO
3	1,2 - Portal Fibrosis	NO
2	1,2 - Portal Fibrosis	NO
1		NO
17		YES
7598		
7	6 - Established Cirrhosis	YES
24	3,4 - Fibrous Speta	NO
3	1,2 - Portal Fibrosis	YES
94340	3,4 - Fibrous Speta	YES
5	0 - No Fibrosis	NO
7	6 - Established Cirrhosis	NO
2	6 - Established Cirrhosis	NO
699		NO
5	6 - Established Cirrhosis	NO
7		NO
290	3,4 - Fibrous Speta	NO
4		NO
		NO
250	6 - Established Cirrhosis	NO
7	6 - Established Cirrhosis	NO
3	6 - Established Cirrhosis	NO
24	6 - Established Cirrhosis	NO
1	6 - Established Cirrhosis	YES
35	3,4 - Fibrous Speta	NO
41	6 - Established Cirrhosis	NO
8	6 - Established Cirrhosis	
1751	0 - No Fibrosis	NO
39	3,4 - Fibrous Speta	NO
2	6 - Established Cirrhosis	YES
1456	6 - Established Cirrhosis	NO
2	3,4 - Fibrous Speta	NO
79		YES
143684	3,4 - Fibrous Speta	
		NO

1836	0 - No Fibrosis	YES
24	6 - Established Cirrhosis	NO
1	1,2 - Portal Fibrosis	NO
5	6 - Established Cirrhosis	NO
1902	6 - Established Cirrhosis	NO
24	6 - Established Cirrhosis	NO
2	1,2 - Portal Fibrosis	YES
11718	6 - Established Cirrhosis	NO
7	6 - Established Cirrhosis	NO
5	6 - Established Cirrhosis	NO
1		NO
4		NO
22		NO
25	1,2 - Portal Fibrosis	NO
8698		NO
419		NO
36		NO
9804		NO
725		NO
27		NO
236	6 - Established Cirrhosis	NO
9		NO
9	6 - Established Cirrhosis	NO
8751	3,4 - Fibrous Speta	NO
2693	6 - Established Cirrhosis	NO
41	6 - Established Cirrhosis	NO
5166	6 - Established Cirrhosis	NO
36	6 - Established Cirrhosis	YES
50149	3,4 - Fibrous Speta	NO
7	3,4 - Fibrous Speta	NO
927	6 - Established Cirrhosis	NO
945	6 - Established Cirrhosis	YES
3	3,4 - Fibrous Speta	YES
10	0 - No Fibrosis	NO
11	1,2 - Portal Fibrosis	NO
44	5 - Nodular Formation and I	YES
3	6 - Established Cirrhosis	YES
4	0 - No Fibrosis	
4	3,4 - Fibrous Speta	YES
3	5 - Nodular Formation and Incomplete Cirrhosis	
10	6 - Established Cirrhosis	
3	0 - No Fibrosis	
2466	3,4 - Fibrous Speta	
1388	1,2 - Portal Fibrosis	NO
8	3,4 - Fibrous Speta	YES
		NO
47	6 - Established Cirrhosis	NO
7	6 - Established Cirrhosis	NO
451		NO
4	1,2 - Portal Fibrosis	NO
2505	6 - Established Cirrhosis	YES
3	1,2 - Portal Fibrosis	
141		YES

vascular infiltration
None
None
None
None
None
Micro
None
None
None
Micro
None
Micro
Micro
None
None
None
None
None
None
None
None

None
None

Micro
Macro
None
None
None
None
None
None
None
None
None
None
None
None
None
Micro
None
None
Micro
Micro
None
None
None
Micro

Micro

Micro
None
Micro
None
Micro
None
Micro
Micro
Micro
Micro
None
None
None
Micro
Macro
None
Macro
None
None
None
Micro
Micro
None
None
None
None
None
None
None
Micro
None
None
None
None
None
None
None
None
None
Micro
None
None
Micro
None
None
None
None
None
None
None
Micro
None
Macro
