# Supplementary material for: Immune-Related lncRNAs with WGCNA Identified the Function of SNHG10 in HBV-Related Hepatocellular Carcinoma
Source: J Oncol. 2022 Jul 6;2022:9332844. doi: 10.1155/2022/9332844 (PMC9279027; doi:10.1155/2022/9332844)
Supplement: Supplementary Materials — Supplementary table 1: immune‐related gene expressions in HBV-related hepatocellular carcinoma from TCGA database for the WGCNA analysis. Supplementary table 2: the clinical characteristics of these eligible patients. Supplementary table 3: list of immune-related genes in the co-expression modules. Supplementary table 4: pathway analysis mapped the identification in the red co-expression module. Supplementary table 5: the co-expression analysis between immune-related genes in the red co-expression module and lncRNAs. Supplementary table 6: 33 immune-related lncRNAs were significant related to the overall survival. Supplementary table 7: lasso regression was constructed examining the relationship between gene signature and HCC risk. Supplementary table 8: quantification of the abundance of immune cell infiltration in tumor microenvironment by CIBERSORT web portal with the LM22 signature. [file 9332844.f1.zip › Supplementary table 3.pdf]

Supplementary table 3: List of immune related genes in the co-ex
List of genes in the co-expression magenta module
CLEC4M
IFI30
PROCR
ADRM1
KIAA0368
TRPC4AP
CD209
UBXN1
ERAP1
TAPBPL
KIR2DL5A
ERAP2
ULBP3
ULBP2
ULBP1
KIR3DL3
RAET1E
RAET1L
UBR1
RAET1G
PDIA2
CD79A
CD79B
LYN
SYK
BTK
BLNK
VAV3
VAV1
VAV2
RAC1
RAC2
RAC3
PPP3CA
PPP3CB
PPP3CC
CHP
PPP3R1
PPP3R2
CHP2
NFAT5
NFATC1
NFATC2
NFATC3
NFATC4
HRAS
KRAS
NRAS
FOS
JUN
CARD11
BCL10

MALT1
CHUK
IKBKB
IKBKG
NFKB1
RELA
NFKBIA
NFKBIB
NFKBIE
CD81
CD19
CR2
PIK3R5
PIK3R1
PIK3R2
PIK3R3
PIK3CA
PIK3CB

:pression modules.
List of genes in the co-expression red module
ACKR2
ACKR4
ADGRE5
AIM2
ANXA11
APLN
APOA1
APOA2
APOA4
APOBEC3F
APOBEC3G
AQP9
ARHGDIB
ATP6V0A2
BCAR1
BCL10
BCL2
BLNK
BNIP3
BNIP3L
BST1
BST2
C1QBP
C2
C5AR1
CADM1
CCL18
CCL19
CCL2
CCL20
CCL21
CCL22
CCL23
CCL24
CCL25
CCL26
CCL27
CCL4
CCL5
CCR1
CCR2
CCR4
CCR5
CCR6
CCR8
CCR9
CD164
CD1D
CD22
CD274
CD28
CD40LG

CD7
CD74
CD79A
CD79B
CD83
CD86
CD96
CEACAM8
CEBPB
CEBPG
CFHR1
CHST4
RPS19
TRAF6
DEFB1
CMKLR1
TARBP2
IL18BP
WAS
CCL25
TCF7
FCGR3B
APOBEC3G
CTLA4
IL16
TCF12
ARHGDIB
IL18
CD24
LCP2
CKLF
CCL4
AQP9
APOA4
IL2RG
LST1
HCLS1
KMT2A
CD3E
CD79B
SIRPG
PTAFR
FCN1
CCR5
MAFB
NCOA6
IFI16
SPI1
CIITA
CTSW
IGSF6
LY86
EBI3
LCK

LTF
CCL26
CD2
GTPBP1
CD79A
HELLS
CXCL13
NCF4
SLA2
CST7
LILRB2
MAP4K1
CD96
NFAM1
CCL5
GZMA
SIT1
ITGB2
SP2
ZAP70
CD86
CTSE
CD3D
S1PR4
IL15
IL16
IL17A
IL17B
IL18
IL18BP
IL1R2
IL2
IL27
IL27RA
IL2RA
IL2RG
IL32
IL4
IL4R
IL6
IL6R
IL6ST
IL7
IL7R
IRF8
KIR2DL1
KIR2DL3
KRT1
LAT
LAT2
LAX1
LCP2
LILRB2
LTB4R

LTF
LY75
LY86
MADCAM1
MALT1
MAP3K7
MAP4K2
MBL2
MBP
MNX1
MR1
MS4A1
MS4A2
NCF4
NCR1
NFAM1
NFIL3
OPRD1
OPRK1
PAX5
PDCD1
POU2AF1
POU2F2
PRELID1
PRKRA
PSMB10
PTAFR
PTGDR2
PTGER4
PTPRC
PYDC1
RAG1
RFX1
RGS1
RSAD2
S1PR4
SECTM1
SEMA3C
SEMA4D
SEMA7A
SFTPD
SKAP1
SLA2
SOCS5
SP2
SPINK5
ST6GAL1
TAPBP
TARBP2
TCF12
TCF7
TENM1
TGFB1
TGFB2

THY1
TLR7
TLR8
TNFAIP1
TNFRSF14
TNFRSF4
TNFSF13
TRAF2
TRAF6
TRAT1
TREM1
TREM2
TRIM22
UBE2N
VIPR1
VTN
WAS
XBP1
YTHDF2
ZAP70
ZEB1
BLM
KCNC1
HIST1H2AL
PTTG2
UGT3A2
LOC399815
HIST1H3C
RAET1K
RRH
NCRNA00189
SH2D6
HIST1H1B
DMC1
RIBC2
C5orf47
HIST1H2BO
LOC127841
LOC339674
FER1L4
HIST1H4C
MYLK2
NUP62CL
PTTG3P
C2orf48
IGF2BP3
DPY19L2P2
RACGAP1P
MYB
WDR93
DHRS2
RDM1
TP73
CLSPN

FAM40B
NEIL3
CFHR3
NEB
E2F7
GPR19
HIF3A
ZNF404
CENPI
FANCB
ACBD7
CFHR4
DUSP9
KIF15
XRCC2
MTCP1
RAD54L
TTC26
MCM10
KIF18A
CCNE1
CENPK
ITPKA
ERCC6L
BFSP1
C15orf42
SGOL1
PRTFDC1
TNFRSF11A
FAM54A
C1orf135
CDCA2
GSG2
PSMC3IP
POLQ
KIAA1524
PIF1
ORC1L
GALNTL2
ABCA9
ESCO2
SPIN4
SPAG8
E2F8
BLM

| List of genes in the co-expression black modul | List of genes in the co-expression brown |
| --- | --- |
| AKT3 | IL16 |
| AKT1 | IL17A |
| AKT2 | IL17B |
| GSK3B | IL17C |
| INPP5D | IL17D |
| CD22 | IL17F |
| CD72 | IL18 |
| PTPN6 | IL19 |
| LILRB3 | IL1A |
| FCGR2B | IL1B |
| RASGRP3 | IL1F10 |
| PLCG2 | IL1F5 |
| PRKCB | IL1F6 |
| IFITM1 | IL1F7 |
| IGHA1 | IL1F8 |
| IGHA2 | IL1F9 |
| IGHD | IL1RN |
| IGHE | IL2 |
| IGHG1 | IL20 |
| IGHG2 | IL21 |
| IGHG3 | IL22 |
| IGHG4 | IL23A |
| IGHM | IL24 |
| IGHV2-70 | IL25 |
| IGHV3-23 | IL26 |
| IGKC | IL27 |
| IGKV1-5 | IL28A |
| IGKV2-40 | IL28B |
| IGKV3-20 | IL29 |
| IGKV3D-11 | IL3 |
| IGKV3D-20 | IL31 |
| IGKV4-1 | IL32 |
| IGLC3 | IL33 |
| IGLV7-43 | IL34 |
| C3 | IL4 |
| C5 | IL5 |
| CAMP | IL6 |
| CCL1 | IL6ST |
| CCL11 | IL7 |
| CCL13 | IL8 |
| CCL14 | IL9 |
| CCL15 | INHA |
| CCL16 | INHBA |
| CCL17 | INHBB |
| CCL18 | INHBC |
| CCL19 | INHBE |
| CCL2 | INS |
| CCL20 | INSL3 |
| CCL21 | INSL4 |
| CCL22 | INSL5 |
| CCL23 | INSL6 |
| CCL24 | JAG1 |

CCL25
CCL26
CCL27
CCL28
CCL3
CCL3L3
CCL4
CCL4L1
CCL5
CCL7
CCL8
CKLF
CMA1
CTSG
CX3CL1
CXCL1
CXCL10
CXCL11
CXCL12
CXCL13
CXCL14
CXCL16
CXCL17
CXCL2
CXCL3
CXCL5
CXCL6
CXCL9
CYR61
DEFA1
DEFA3
DEFA5
DEFB1
DEFB104A
DEFB4
EDN1
EDN2
EDN3
FGF10
FGF2
HTN3
IL8
LECT2
PF4
PF4V1
PLAU
PPBP
PROK2
JAG2
KGFLP1
KGFLP2
KITLG
KL
LACRT
LECT2
LEFTY1
LEFTY2
LEP
LHB

List of genes in the co-expression blue module
CTNND2
DLK1
SLC6A11
HEPN1
TUBBP5
OXT
FER1L6
MYH4
TRIM71
IGFBPL1
B4GALNT2
MCCD1
LOC146336
C9orf122
DIO3
HS6ST2
CCDC64B
PRAME
ESRP1
PRSS16
LOC284749
SCGB2A1
LYPD6B
TRHDE
SFRP5
TRPV6
HOXC9
TNNT1
PDX1
GDF10
DQX1
B4GALNT4
GPRIN2
KISS1R
KCNS1
LRRC26
CKMT1A
STK33
OVOL2
FAM169B
FIBCD1
HOXC6
HMGA2
FAM83F
C4orf6
PNCK
KCNK15
ATP10B
IL20RA
SLC6A19
OVOL1
LOC285629

GABRB3
DMBT1
CLDN18
MAP7D2
ACIN1
ACKR2
ACKR4
ACVR1B
ACVR2A
ADGRE5
AIM2
AIMP1
AKT1
ALAS2
ANXA11
APLN
APOA1
APOA2
APOA4
APOBEC3F
APOBEC3G
AQP9
ARHGDIB
ATP6V0A2
AZU1
BCAR1
BCL10
BCL2
BLNK
BNIP3
BNIP3L
BST1
BST2
C1QBP
C2
C5AR1
CADM1
CALCA
CARTPT
CCL18
CCL19
CCL2
CCL20
CCL21
CCL22
CCL23
CCL24
CCL25
CCL26
CCL27
CCL4
CCL5
CCR1
CCR2

CCR4
CCR5
CCR6
CCR8
CCR9
CD164
CD1D
CD2
CD22
CD24
CD274
CD276
CD28
CD34
CD3D
CD3E
CD4
CD40LG
CD47
CD7
CD74
CD79A
CD79B
CD83
CD86
CD96
CDC42
CDK6
CEACAM8
CEBPB
CEBPG
CFHR1
CHST4
CHUK
CIITA
CKLF
CLEC7A
CMKLR1
CNIH1
CNR2
COLEC12
CRHR1
CRTAM
CSF1
CST7
CTLA4
CTSC
CTSE
CTSG
CTSS
CTSW
CX3CL1
CXCL12
CXCL13

CXCL8
CXCR2
CXCR4
DCSTAMP
DEFA1
DEFB1
DEFB103A
DEFB118
DEFB127
DEFB4A
DMBT1
DOCK2
DPP4
DPP8
DYRK3
EBI3
ELF4
ELP1
ERAP2
EREG
ETS1
FCAR
FCGR1A
FCGR2B
FCGR3A
FCGR3B
FCGRT
FCN1
FCN2
FOXO3
FOXP3
FTH1
FYB1
FYN
GBP2
GEM
GLMN
GPI
GPR183
GPR65
GTPBP1
GZMA
HAMP
HCLS1
HDAC4
HDAC5
HDAC7
HDAC9
HELLS
HLA-DRB3
HRH2
ICOSLG
IFI16
IFI6

IFITM2
IFITM3
IFNK
IFNL1
IFNLR1
IGSF6
IK
IKBKG
IL10
IL10RB
IL12A
IL12B
IL15
IL16
IL17A
IL17B
IL18
IL18BP
IL1R2
IL2
IL21
IL27
IL27RA
IL2RA
IL2RG
IL31RA
IL32
IL4
IL4R
IL6
IL6R
IL6ST
IL7
IL7R
INHA
INHBA
INS
IRF8
ITGB2
JAG2
KAT6A
KAT8
KIR2DL1
KIR2DL3
KIRREL3
KMT2A
KRT1
LAT
LAT2
LAX1
LCK
LCP2
LDB1
LIG1

LIG3
LILRB2
LRMP
LST1
LTB4R
LTF
LY75
LY86
LYN
MADCAM1
MAFB
MAL
MALT1
MAP3K7
MAP4K1
MAP4K2
MBL2
MBP
MIA3
MLF1
MMP9
MNX1
MR1
MS4A1
MS4A2
MYH9
NCF4
NCK1
NCK2
NCOA6
NCR1
NFAM1
NFIL3
NHEJ1
NLRC3
NOTCH2
NOTCH4
OPRD1
OPRK1
PAX5
PDCD1
PF4
POU2AF1
POU2F2
PRELID1
PREX1
PRG3
PRKRA
PRL
PSMB10
PTAFR
PTGDR2
PTGER4
PTPRC

PYDC1
RAB3D
RAG1
RASGRP4
RFX1
RGS1
RPS19
RSAD2
RUNX1
S1PR4
SAA1
SART1
SCG2
SCIN
SECTM1
SEMA3C
SEMA4D
SEMA7A
SFTPD
SIRPG
SIT1
SKAP1
SLA2
SNRK
SOCS5
SOD1
SP2
SPACA3
SPI1
SPINK5
ST6GAL1
SYK
TAPBP
TARBP2
TAZ
TBX1
TCF12
TCF7
TENM1
TGFB1
TGFB2
THY1
TLR4
TLR7
TLR8
TNFAIP1
TNFRSF14
TNFRSF4
TNFSF13
TPD52
TRAF2
TRAF6
TRAT1
TREM1

TREM2
TRIM22
UBE2N
VIPR1
VTN
WAS
XBP1
YTHDF2
ZAP70
ZBTB16
ZEB1
ZNF675

List of genes in the co-expression turqoise module
MAP2K1
MAP2K2
MAPK1
MAPK3
TNF
CSF2
IFNG
KIR2DS1
KIR2DS3
KIR2DS4
KIR2DS5
NCR2
TYROBP
LCK
FCGR3A
FCGR3B
NCR1
NCR3
FCER1G
CD247
ZAP70
SYK
LCP2
LAT
PLCG1
PLCG2
SH3BP2
PIK3CA
PIK3CB
PIK3CD
PIK3CG
PIK3R5
PIK3R1
PIK3R2
PIK3R3
FYN
SHC2
SHC4
SHC3
SHC1
GRB2
SOS1
SOS2
HRAS
KRAS
NRAS
ARAF
BRAF
RAF1
MICA
MICB
ULBP3

ULBP2
ULBP1
KLRK1
HCST
CD48
CD244
PPP3CA
PPP3CB
PPP3CC
CHP
PPP3R1
PPP3R2
CHP2
NFAT5
NFATC1
NFATC2
NFATC3
NFATC4
PRKCA
PRKCB
PRKCG
SH2D1B
SH2D1A
IFNGR1
IFNGR2
IFNA2
IFNA4
IFNA5
IFNA6
IFNA7
IFNA8
IFNA10
IFNA13
IFNA14
IFNA16
IFNA17
IFNA21
IFNB1
IFNAR1
IFNAR2
TNFSF10
TNFRSF10D
TNFRSF10C
TNFRSF10B
TNFRSF10A
FASLG
FAS
GZMB
PRF1
CASP3
BID
CD3D
CD3E
CD3G

CD247
CD4
CD8A
CD8B
PTPRC
LCK
FYN
ZAP70
LCP2
LAT
ITK
TEC
NCK1
NCK2
VAV3
VAV1
VAV2
GRAP2
GRB2
PAK1
PAK2
PAK3
PAK4
PAK6
PAK7
RHOA
CDC42
PPP3CA
PPP3CB
PPP3CC
CHP
PPP3R1
PPP3R2
CHP2
NFAT5
NFATC1
NFATC2
NFATC3
NFATC4
SOS1
SOS2
HRAS
KRAS
NRAS
FOS
JUN
CARD11
BCL10
MALT1
CHUK
IKBKB
IKBKG
NFKB1
RELA

NFKBIA

List of genes in the co-expression yellow module
NFKBIB
NFKBIE
CD28
ICOS
CD40LG
PIK3R5
PIK3R1
PIK3R2
PIK3R3
PIK3CA
PIK3CB
PIK3CD
PIK3CG
AKT3
AKT1
AKT2
MAP3K8
MAP3K14
PDCD1
CTLA4
PTPN6
CBLC
CBL
CBLB
IL2
IL4
IL5
IL10
IFNG
CSF2
TNF
CDK4
RASGRP1
PDK1
PLCG1
PRKCQ
TRBC1
TRBV12-3
TRGV3
BMP1
BMP10
BMP15
BMP2
BMP3
BMP4
BMP5
BMP6
BMP7
BMP8A
BMP8B
GDF1
GDF10

GDF11
GDF15
GDF2
GDF3
GDF5
GDF6
GDF7
GDF9
GDNF
INHA
INHBA
INHBB
INHBC
INHBE
LEFTY1
LEFTY2
NODAL
TGFB1
TGFB2
TGFB3
ACVR1B
ACVR1C
ACVR2A
ACVR2B
ACVRL1
AMHR2
BMPR1A
BMPR1B
BMPR2
TGFBR1
TGFBR2
TGFBR3
TNFRSF11B
TNFSF10
TNFSF11
TNFSF12
TNFSF13
TNFSF13B
TNFSF14
TNFSF15
TNFSF18
TNFSF4
TNFSF8
TNFSF9
TNFRSF10B
TNFRSF10C
TNFRSF10D
TNFRSF11A
TNFRSF12A
TNFRSF13B
TNFRSF13C
TNFRSF14
TNFRSF17
TNFRSF18

TNFRSF19
TNFRSF1A
TNFRSF1B
TNFRSF21
TNFRSF25
TNFRSF4
TNFRSF6B
TNFRSF8
TNFRSF9

List of genes in the co-expression pink module
CCR1
CCR10
CCR3
CCR4
CCR5
CCR6
CCR7
CCR8
CCR9
CCRL1
CCRL2
CMKLR1
CX3CR1
CXCR3
CXCR4
CXCR5
CXCR6
CXCR7
CYSLTR1
CYSLTR2
DARC
EDNRA
EDNRB
FPR1
FPR2
GPR17
GPR32
GPR33
GPR44
GPR77
IL8RA
IL8RB
LTB4R
LTB4R2
PLAUR
PLXNA1
PLXNA2
PLXNA3
PLXNA4
PLXNB1
PLXNB2
PLXNB3
PLXNC1
PLXND1
PTAFR
ROBO1
ROBO2
ROBO3
RXFP3
XCR1
ADIPOQ
ADM

ADM2
AGRP
AGT
AMBN
AMELX
AMH
ANGPTL5
ANGPTL7
APLN
AREG
ARMET
ARMETL1
ARTN
AVP
AZU1
BDNF
BMP1
BMP10
BMP15
BMP2
BMP3
BMP4
BMP5
BMP6
BMP7
BMP8A
BMP8B
BTC
C19orf10
C3
C5
CALCA
CALCB
CAMP
CAT
CCK
CCL1
CCL11
CCL13
CCL14
CCL15
CCL16
CCL17
CCL18
CCL19
CCL2
CCL20
CCL21
CCL22
CCL23
CCL24
CCL25
CCL26
CCL27

CCL28
CCL3
CCL3L3
CCL4
CCL4L1
CCL5
CCL7
CCL8
