# Supplementary material for: Immune-Related lncRNAs with WGCNA Identified the Function of SNHG10 in HBV-Related Hepatocellular Carcinoma
Source: J Oncol. 2022 Jul 6;2022:9332844. doi: 10.1155/2022/9332844 (PMC9279027; doi:10.1155/2022/9332844)
Supplement: Supplementary Materials — Supplementary table 1: immune‐related gene expressions in HBV-related hepatocellular carcinoma from TCGA database for the WGCNA analysis. Supplementary table 2: the clinical characteristics of these eligible patients. Supplementary table 3: list of immune-related genes in the co-expression modules. Supplementary table 4: pathway analysis mapped the identification in the red co-expression module. Supplementary table 5: the co-expression analysis between immune-related genes in the red co-expression module and lncRNAs. Supplementary table 6: 33 immune-related lncRNAs were significant related to the overall survival. Supplementary table 7: lasso regression was constructed examining the relationship between gene signature and HCC risk. Supplementary table 8: quantification of the abundance of immune cell infiltration in tumor microenvironment by CIBERSORT web portal with the LM22 signature. [file 9332844.f1.zip › Supplementary table 4.pdf]

Supplementary table 4:Pathway analysis mapped the
Category

The co-expression red module

e identifed in the red co-expression module.

Term	P-Value
Cytokine-cytokine receptor interaction	2.8E-32
Intestinal immune network for IgA production	1.6E-15
Chemokine signaling pathway	3.5E-14
T cell receptor signaling pathway	1.3E-12
NF-kappa B signaling pathway	1.6E-11
Jak-STAT signaling pathway	0.000000016
Staphylococcus aureus infection	0.000003
Toll-like receptor signaling pathway	0.0000051
TNF signaling pathway	0.0000097
NOD-like receptor signaling pathway	0.000049
B cell receptor signaling pathway	0.000081
RIG-I-like receptor signaling pathway	0.0004

Adjust P value
3.60E-30
1E-13
1.5E-12
4.1E-11
4E-10
0.00000023
0.000024
0.000038
0.000066
0.00032
0.00045
0.0022
