# Supplementary material for: Immune-Related lncRNAs with WGCNA Identified the Function of SNHG10 in HBV-Related Hepatocellular Carcinoma
Source: J Oncol. 2022 Jul 6;2022:9332844. doi: 10.1155/2022/9332844 (PMC9279027; doi:10.1155/2022/9332844)
Supplement: Supplementary Materials — Supplementary table 1: immune‐related gene expressions in HBV-related hepatocellular carcinoma from TCGA database for the WGCNA analysis. Supplementary table 2: the clinical characteristics of these eligible patients. Supplementary table 3: list of immune-related genes in the co-expression modules. Supplementary table 4: pathway analysis mapped the identification in the red co-expression module. Supplementary table 5: the co-expression analysis between immune-related genes in the red co-expression module and lncRNAs. Supplementary table 6: 33 immune-related lncRNAs were significant related to the overall survival. Supplementary table 7: lasso regression was constructed examining the relationship between gene signature and HCC risk. Supplementary table 8: quantification of the abundance of immune cell infiltration in tumor microenvironment by CIBERSORT web portal with the LM22 signature. [file 9332844.f1.zip › Supplementary table 5.pdf]

Supplementary table 5: The coexpression analysis between immune related g

Immunegen	lncRNA	cor	pvalue	Regulation
RPS19	AC112491.1	0.7006259	6.27E-17	postive
TRAF6	SEMA6A-AS11	0.70109778	5.86E-17	postive
DEFB1	LINC01671	0.70265854	4.67E-17	postive
CMKLR1	AC138207.5	0.70429267	3.68E-17	postive
TARBP2	LINC01503	0.70453312	3.55E-17	postive
IL18BP	HLA-DQB1-AS1	0.70473781	3.45E-17	postive
WAS	HLA-DQB1-AS1	0.70506308	3.28E-17	postive
CCL25	AL135924.2	0.70591832	2.90E-17	postive
TCF7	AC008549.1	0.70616854	2.79E-17	postive
FCGR3B	LINC01139	0.70780585	2.19E-17	postive
APOBEC3G	LINC01871	0.70835492	2.02E-17	postive
CTLA4	HLA-DQB1-AS1	0.70906097	1.81E-17	postive
IL16	HLA-DQB1-AS1	0.70933307	1.74E-17	postive
TCF12	MIR31HG	0.70935016	1.74E-17	postive
ARHGDIB	HLA-DQB1-AS1	0.71013913	1.54E-17	postive
IL18	AC138207.5	0.71063536	1.43E-17	postive
CD24	SNHG12	0.71131999	1.29E-17	postive
LCP2	HLA-DQB1-AS1	0.71145153	1.27E-17	postive
CKLF	AC016405.3	0.71152075	1.25E-17	postive
CCL4	LINC01871	0.71273066	1.04E-17	postive
AQP9	LINC01278	0.71314786	9.79E-18	postive
CTLA4	AC004687.1	0.71387499	8.76E-18	postive
APOA4	AL138826.1	0.71409093	8.48E-18	postive
IL2RG	PCED1B-AS1	0.71448563	7.98E-18	postive
RPS19	PRR34-AS1	0.71663945	5.73E-18	postive
LST1	AC138207.5	0.71736759	5.12E-18	postive
HCLS1	HLA-DQB1-AS1	0.71747628	5.03E-18	postive
KMT2A	MIR31HG	0.71790355	4.71E-18	postive
RPS19	AP000240.1	0.71792556	4.70E-18	postive
CD3E	LINC01871	0.71802513	4.62E-18	postive
CD79B	AC110741.1	0.71925852	3.81E-18	postive
SIRPG	LINC01871	0.71990175	3.45E-18	postive
LST1	HLA-DQB1-AS1	0.7208027	2.99E-18	postive
CKLF	AL138724.1	0.72399884	1.80E-18	postive
CKLF	AC006205.2	0.72480286	1.59E-18	postive
PTAFR	PCED1B-AS1	0.72484369	1.58E-18	postive
KMT2A	ANKRD10-IT1	0.72502476	1.53E-18	postive
CKLF	LINC02027	0.72521643	1.48E-18	postive
CCL25	AC147651.3	0.725814	1.35E-18	postive
FCN1	HLA-DQB1-AS1	0.72652797	1.20E-18	postive
CCR5	HLA-DQB1-AS1	0.72661743	1.19E-18	postive
MAFB	AC254562.3	0.72763681	1.00E-18	postive
NCOA6	AC005288.1	0.72912382	7.89E-19	postive
IFI16	AC110741.1	0.72927629	7.70E-19	postive
SPI1	PCED1B-AS1	0.73003151	6.80E-19	postive
RPS19	AL355353.1	0.73366021	3.73E-19	postive
CD24	AC026740.1	0.73480961	3.08E-19	postive
CIITA	MMP25-AS1	0.73515678	2.91E-19	postive
CTSW	HLA-DQB1-AS1	0.73521726	2.88E-19	postive

IGSF6	LINC01871	0.73606589	2.50E-19	postive
ARHGDIB	AC004687.1	0.73710865	2.09E-19	postive
FCGR3B	AC243836.1	0.74214105	8.85E-20	postive
LY86	AC138207.5	0.74337789	7.14E-20	postive
CKLF	AL121832.2	0.74410063	6.29E-20	postive
APOBEC3G	PCED1B-AS1	0.74507736	5.30E-20	postive
CCR5	AC004687.1	0.74592786	4.57E-20	postive
CKLF	AC008035.1	0.74614941	4.39E-20	postive
EBI3	PCED1B-AS1	0.74655572	4.09E-20	postive
LCK	LINC01871	0.74755392	3.43E-20	postive
LTF	AC025575.2	0.74977628	2.30E-20	postive
HCLS1	AC004687.1	0.75078554	1.92E-20	postive
CD79B	AC105118.1	0.75448972	9.80E-21	postive
CCL26	C1QTNF1-AS1	0.75450314	9.77E-21	postive
CD2	LINC01871	0.75559716	7.99E-21	postive
GTPBP1	Z97056.1	0.75658987	6.65E-21	postive
LCP2	PCED1B-AS1	0.75677638	6.43E-21	postive
LY86	LINC01871	0.75770604	5.41E-21	postive
SPI1	AC138207.5	0.75863423	4.55E-21	postive
CD79A	AC004687.1	0.76101606	2.90E-21	postive
CKLF	LINC00239	0.76202945	2.40E-21	postive
CD79B	Z98257.1	0.76210199	2.36E-21	postive
CD79A	PCED1B-AS1	0.76250875	2.19E-21	postive
HELLS	AC099850.3	0.76303916	1.98E-21	postive
MAFB	MALAT1	0.76336066	1.86E-21	postive
CXCL13	AC098487.1	0.76360975	1.77E-21	postive
NCF4	PCED1B-AS1	0.76362991	1.76E-21	postive
CKLF	AC109322.1	0.76396385	1.65E-21	postive
SLA2	AC004687.1	0.76457393	1.47E-21	postive
CMKLR1	PCED1B-AS1	0.76537505	1.26E-21	postive
LST1	LINC1278	0.76689361	9.39E-22	postive
SIRPG	HLA-DQB1-AS1	0.76720518	8.83E-22	postive
IGSF6	PCED1B-AS1	0.76839908	6.99E-22	postive
APOA4	AGAP1-IT1	0.76972495	5.39E-22	postive
CXCL13	AC243836.1	0.77063308	4.50E-22	postive
EBI3	AC138207.5	0.77072064	4.42E-22	postive
CST7	LINC01871	0.77118874	4.03E-22	postive
LILRB2	PCED1B-AS1	0.7715763	3.73E-22	postive
MAP4K1	HLA-DQB1-AS1	0.77377245	2.40E-22	postive
CD96	PCED1B-AS1	0.77386367	2.36E-22	postive
NFAM1	PCED1B-AS1	0.77431371	2.15E-22	postive
FCGR3B	AC098487.1	0.77441232	2.11E-22	postive
SIRPG	AC004687.1	0.77609915	1.50E-22	postive
CCL4	PCED1B-AS1	0.77784111	1.05E-22	postive
MAFB	LINC00460	0.7778886	1.04E-22	postive
CCL5	HLA-DQB1-AS1	0.77859027	8.97E-23	postive
IL16	AC004687.1	0.77893567	8.35E-23	postive
CKLF	SNHG6	0.77960196	7.27E-23	postive
GZMA	HLA-DQB1-AS1	0.78070673	5.77E-23	postive
SIT1	HLA-DQB1-AS1	0.78127085	5.13E-23	postive
CD2	HLA-DQB1-AS1	0.78228883	4.14E-23	postive

CTSW	AC004687.1	0.78286874	3.66E-23	postive
CD3E	HLA-DQB1-AS1	0.78302047	3.55E-23	postive
ITGB2	PCED1B-AS1	0.7889609	9.89E-24	postive
CST7	HLA-DQB1-AS1	0.79073384	6.70E-24	postive
WAS	AC004687.1	0.7907945	6.61E-24	postive
SP2	LBX2-AS1	0.79132237	5.88E-24	postive
CKLF	SNHG19	0.79629816	1.92E-24	postive
IFI16	AC105118.1	0.79761676	1.42E-24	postive
FCGR3B	AC026202.2	0.79936032	9.53E-25	postive
AQP9	LINC01124	0.80037366	7.53E-25	postive
LTF	AC110741.1	0.80110306	6.35E-25	postive
ZAP70	HLA-DQB1-AS1	0.8018119	5.38E-25	postive
SLA2	HLA-DQB1-AS1	0.80452722	2.83E-25	postive
LY86	PCED1B-AS1	0.80515099	2.44E-25	postive
LILRB2	HLA-DQB1-AS1	0.80541205	2.29E-25	postive
CKLF	AL391056.1	0.80671643	1.67E-25	postive
IL18BP	DLGAP1-AS5	0.810482	6.68E-26	postive
LCK	HLA-DQB1-AS1	0.81074659	6.26E-26	postive
CCL4	HLA-DQB1-AS1	0.81240911	4.14E-26	postive
IL16	PCED1B-AS1	0.81559422	1.85E-26	postive
CTSW	LINC01871	0.81627291	1.56E-26	postive
CTLA4	PCED1B-AS1	0.82091487	4.68E-27	postive
GZMA	AC004687.1	0.82266221	2.95E-27	postive
CST7	AC004687.1	0.82278247	2.86E-27	postive
CCL5	AC004687.1	0.82474259	1.69E-27	postive
CD86	PCED1B-AS1	0.82723175	8.60E-28	postive
CIITA	DLGAP1-AS5	0.82834446	6.33E-28	postive
CCR5	PCED1B-AS1	0.82836756	6.29E-28	postive
CXCL13	AC026202.2	0.83040803	3.57E-28	postive
NCF4	AC138207.5	0.83681053	5.75E-29	postive
CCL25	AF124730.1	0.83774502	4.37E-29	postive
CD79B	LINC01503	0.8392096	2.84E-29	postive
LCK	LBX2-AS1	0.84348543	7.85E-30	postive
MAP4K1	CTP!-AS2	0.84466135	5.47E-30	postive
ZAP70	AC004687.1	0.84496744	4.98E-30	postive
CTSE	AL133520.1	0.84523503	4.59E-30	postive
SIRPG	PCED1B-AS1	0.84712091	2.55E-30	postive
FCN1	AC004687.1	0.85021297	9.58E-31	postive
FCN1	PCED1B-AS1	0.85042332	8.96E-31	postive
GTPBP1	AL021707.8	0.85173896	5.86E-31	postive
CXCL13	SLC6A1-AS1	0.85209701	5.22E-31	postive
LTF	AC105118.1	0.8523484	4.81E-31	postive
IFI16	Z98257.1	0.85240435	4.72E-31	postive
SLA2	PCED1B-AS1	0.85334385	3.48E-31	postive
CD3D	AC004687.1	0.8535737	3.22E-31	postive
CCL5	LINC01871	0.8539147	2.88E-31	postive
ZAP70	PCED1B-AS1	0.85563874	1.63E-31	postive
IFI16	LINC01503	0.8611172	2.52E-32	postive
CD3D	LINC01871	0.86224772	1.70E-32	postive
HCLS1	PCED1B-AS1	0.86662123	3.57E-33	postive
CCL25	MAFA-AS1	0.86738152	2.71E-33	postive

ARHGDIB	PCED1B-AS1	0.86798891	2.17E-33	postive
SIT1	AC004687.1	0.87217197	4.54E-34	postive
CD3E	AC004687.1	0.87272259	3.68E-34	postive
WAS	PCED1B-AS1	0.87626854	9.31E-35	postive
CST7	PCED1B-AS1	0.87852584	3.79E-35	postive
LST1	LINC01871	0.88235947	7.93E-36	postive
FCGR3B	SLC6A1-AS1	0.88481806	2.82E-36	postive
MAP4K1	PCED1B-AS1	0.88503841	2.57E-36	postive
LTF	Z98257.1	0.88651902	1.36E-36	postive
CD2	AC004687.1	0.88797341	7.23E-37	postive
CTSW	PCED1B-AS1	0.88880593	5.02E-37	postive
GZMA	LINC01871	0.89009662	2.83E-37	postive
FCGR3B	AC015689.1	0.89706604	1.13E-38	postive
LST1	PCED1B-AS1	0.89988397	2.87E-39	postive
SIT1	PCED1B-AS1	0.90674876	8.54E-41	postive
CCL5	PCED1B-AS1	0.90861667	3.13E-41	postive
CXCL13	AC015689.1	0.90872687	2.95E-41	postive
GZMA	PCED1B-AS1	0.91518558	7.68E-43	postive
S1PR4	AC004687.1	0.91830979	1.18E-43	postive
LCK	PCED1B-AS1	0.92064912	2.78E-44	postive
S1PR4	PCED1B-AS1	0.92184852	1.30E-44	postive
CD3E	PCED1B-AS1	0.92287231	6.71E-45	postive
LTF	LINC01503	0.92684997	4.74E-46	postive
CD2	PCED1B-AS1	0.94058423	1.36E-50	postive
CD3D	PCED1B-AS1	0.94393216	7.27E-52	postive
CXCL13	PITPNM2-AS1	0.97991216	1.20E-74	postive
FCGR3B	PITPNM2-AS1	0.99706193	7.13E-118	postive

enes in the red co-expression module and lncRNAs.
