# Supplementary material for: Immune-Related lncRNAs with WGCNA Identified the Function of SNHG10 in HBV-Related Hepatocellular Carcinoma
Source: J Oncol. 2022 Jul 6;2022:9332844. doi: 10.1155/2022/9332844 (PMC9279027; doi:10.1155/2022/9332844)
Supplement: Supplementary Materials — Supplementary table 1: immune‐related gene expressions in HBV-related hepatocellular carcinoma from TCGA database for the WGCNA analysis. Supplementary table 2: the clinical characteristics of these eligible patients. Supplementary table 3: list of immune-related genes in the co-expression modules. Supplementary table 4: pathway analysis mapped the identification in the red co-expression module. Supplementary table 5: the co-expression analysis between immune-related genes in the red co-expression module and lncRNAs. Supplementary table 6: 33 immune-related lncRNAs were significant related to the overall survival. Supplementary table 7: lasso regression was constructed examining the relationship between gene signature and HCC risk. Supplementary table 8: quantification of the abundance of immune cell infiltration in tumor microenvironment by CIBERSORT web portal with the LM22 signature. [file 9332844.f1.zip › Supplementary table 6.pdf]

Supplementary table 6: 33 immune-related lncRNAs were

LncRNA	p value
A1BG-AS1	0.012
AC006942.1	0.031
CTBP-AS2	0.022
SNHG10	0.003
AC012146.1	0.021
HAND2-AS1	0.004
AC016044.1	0.024
AC021074.3	0.023
AC025171.1	0.018
LINC00514	0.018
MALAT1	0.002
AC083809.1	0.019
LINC00205	0.014
AC090152.1	0.008
LBX2-AS1	0.016
LINC00460	0.012
AC100847.1	0.028
AC105942.1	0.014
LINC00844	0.026
ADORA2A-AS1	0.02
AL031316.1	0.025
MIR31HG	0.024
AL133243.2	0.025
AL157373.2	0.008
SEMA6A-AS1	0.01
AL355574.1	0.014
AL360181.1	0.026
AL603839.3	0.02
AP003119.1	0.025
LINC1278	0.024
AP003469.2	0.025
C5orf56	0.008
CARD8-AS1	0.01

e significant related with the overall survival.

Hazard ratio
0.123(0.024-0.634)
2.730(1.098-6.787)
2.449(1.136-5.277)
1.671(1.188-2.352)
1.992(1.108-3.579)
0.379(0.194-0.739)
0.453(0.227-0.903)
0.700(0.515-0.951)
2.554(1.174-5.556)
3.651(1.247-10.689)
4.252(1.674-10.801
1.318(1.047-1.658)
2.301(1.186-4.462)
1.770(1.162-2.697)
3.828(1.278-11.468)
5.177(1.434-18.686)
2.678(1.110-6.460)
1.714(1.116-2.631)
0.271(0.086-0.853)
0.561(0.345-0.912)
1.394(1.042-1.864)
0.178(0.039-0.799)
4.320(1.206-15.471)
1.699(1.148-2.516)
2.586(1.253-5.337)
2.713(1.221-6.024)
0.549(0.326-0.926)
2.306(1.218-4.365)
1.964(1.169-3.299)
4.729(1.643-13.612)
1.837(1.095-3.082)
10.394(2.888-37.408)
3.158(1.153-8.646)
