# Supplementary material for: Immune-Related lncRNAs with WGCNA Identified the Function of SNHG10 in HBV-Related Hepatocellular Carcinoma
Source: J Oncol. 2022 Jul 6;2022:9332844. doi: 10.1155/2022/9332844 (PMC9279027; doi:10.1155/2022/9332844)
Supplement: Supplementary Materials — Supplementary table 1: immune‐related gene expressions in HBV-related hepatocellular carcinoma from TCGA database for the WGCNA analysis. Supplementary table 2: the clinical characteristics of these eligible patients. Supplementary table 3: list of immune-related genes in the co-expression modules. Supplementary table 4: pathway analysis mapped the identification in the red co-expression module. Supplementary table 5: the co-expression analysis between immune-related genes in the red co-expression module and lncRNAs. Supplementary table 6: 33 immune-related lncRNAs were significant related to the overall survival. Supplementary table 7: lasso regression was constructed examining the relationship between gene signature and HCC risk. Supplementary table 8: quantification of the abundance of immune cell infiltration in tumor microenvironment by CIBERSORT web portal with the LM22 signature. [file 9332844.f1.zip › Supplementary table 7.pdf]

Supplementary table 7: Lasso regression was constructed examining the relationship betwe

id	futime	fustat	HAND2-A	LINC0084	SNHG10	MALAT1	LINC0046
TCGA-DD-	0.024658	0	5.488987	4.942339	5.734072	6.418455	6.233542
TCGA-DD-	0.043836	1	5.086645	4.420035	5.345145	6.756209	5.912736
TCGA-G3-	0.073973	1	4.714085	4.909053	4.855418	5.685242	6.608918
TCGA-BC-	0.249315	1	6.024566	5.50946	6.617806	7.126636	6.76604
TCGA-QA-	0.257534	0	6.020467	5.690776	5.420007	5.695042	6.775527
TCGA-DD-	0.315068	1	5.627415	5.586319	5.303297	5.572877	6.362012
TCGA-DD-	0.375342	0	5.516721	5.04679	4.88591	5.486244	6.48537
TCGA-DD-	0.465753	0	5.778103	5.344724	5.397479	5.721216	6.402636
TCGA-DD-	0.534247	1	5.638384	5.705247	6.847672	6.537187	6.642911
TCGA-UB-	0.586301	1	6.095152	5.18131	5.983797	6.50589	6.476311
TCGA-DD-	0.610959	1	5.10253	4.69821	5.840106	6.585946	5.800904
TCGA-G3-	0.983562	1	5.371785	4.723641	5.301474	6.243307	6.127427
TCGA-G3-	0.989041	0	5.934017	5.8733	6.507891	6.932761	6.574412
TCGA-DD-	1.021918	1	5.773481	5.423322	6.132976	6.635177	6.532024
TCGA-DD-	1.043836	1	5.369692	5.231394	5.745827	6.519411	5.625349
TCGA-DD-	1.136986	1	5.778195	5.528188	6.369772	6.662698	6.525894
TCGA-DD-	1.164384	1	5.937116	5.577589	6.740173	6.400534	6.678552
TCGA-DD-	1.183562	1	5.54092	5.28526	5.24966	6.710947	6.232186
TCGA-G3-	1.238356	1	6.023869	5.083099	6.679187	6.870045	6.170819
TCGA-DD-	1.241096	0	5.921591	5.131529	6.143852	6.559177	6.114929
TCGA-DD-	1.284932	1	5.922852	5.720591	6.276976	6.58115	6.562681
TCGA-DD-	1.309589	0	5.228204	5.262008	5.778695	5.778695	5.731159
TCGA-G3-	1.315068	0	5.741701	5.591982	5.977014	6.695799	6.200492
TCGA-UB-	1.369863	0	5.917536	5.527667	4.998137	6.374121	6.30647
TCGA-K7-	1.40274	0	5.214431	5.34954	5.545056	6.44083	5.783986
TCGA-DD-	1.520548	0	5.955096	5.425438	6.929826	7.09118	6.509656
TCGA-G3-	1.60274	0	5.257997	4.665329	5.562945	6.01287	5.976364
TCGA-DD-	1.608219	0	5.891579	4.825566	6.86178	6.944607	6.555914
TCGA-DD-	1.665753	0	5.713855	5.575179	5.697362	6.326184	6.313705
TCGA-DD-	1.742466	0	5.733628	5.489646	5.691915	6.343376	6.302378
TCGA-RC-	1.753425	0	5.95393	5.080307	5.688125	6.36195	6.581917
TCGA-XR-	1.89863	1	5.662101	4.54015	5.688105	5.811629	6.064906
TCGA-ZP-	1.934247	0	5.812765	5.349147	6.286445	6.670813	6.446043
TCGA-DD-	1.978082	0	4.446955	5.241987	5.976393	5.195837	6.145884
TCGA-G3-	2.136986	0	5.768689	5.110452	6.062564	6.709392	6.157602
TCGA-DD-	2.219178	0	5.369546	5.48958	5.423353	6.145212	6.279151
TCGA-DD-	2.460274	0	5.893712	5.852219	5.924388	6.120317	6.33351
TCGA-DD-	2.873973	0	5.383542	4.868094	6.428547	6.610636	6.08878
TCGA-DD-	3.136986	0	4.85459	5.102441	3.900887	5.708885	5.775411
TCGA-DD-	3.40274	0	5.971831	5.264816	6.519267	6.612319	6.607139
TCGA-DD-	3.684932	0	5.838829	5.714393	6.759372	6.49671	6.484849
TCGA-DD-	4.443836	1	5.217759	5.013591	6.273029	6.461477	6.20028
TCGA-G3-	4.482192	0	5.946828	5.578501	4.818549	6.419515	6.505826
TCGA-DD-	4.616438	1	5.842533	4.944043	6.305298	6.691761	6.383795
TCGA-G3-	4.873973	0	5.871567	5.550449	6.85576	6.96233	6.542569
TCGA-DD-	4.942466	0	5.963319	5.549063	6.044418	7.006703	6.434098
TCGA-DD-	6.30411	0	5.572766	5.124104	5.305378	5.718791	6.385074
TCGA-DD-	6.30411	0	5.608768	4.961405	4.138715	5.965839	6.433299
TCGA-DD-	6.367123	0	5.752302	5.295068	6.198549	6.174328	6.234783

TCGA-DD-	6.616438	0	5.58704	5.027676	5.604789	6.44963	6.26921
TCGA-DD-	6.726027	0	5.866755	5.358629	6.290518	6.873176	6.039266
TCGA-DD-	0.032877	1	6.233542	6.598036	5.286043	5.696466	5.574801
TCGA-5C-	0.054795	0	5.912736	6.060228	5.765107	5.82069	5.528821
TCGA-DD-	0.50137	0	6.608918	5.641854	5.558546	5.888259	5.774627
TCGA-DD-	0.950685	0	6.76604	6.683665	5.3745	5.891648	4.626664
TCGA-DD-	1.194521	0	6.775527	6.594409	4.80812	5.768344	5.447231
TCGA-DD-	1.254795	0	6.362012	6.026145	5.31301	5.798306	5.617917
TCGA-RC-	1.282192	0	6.48537	6.076888	5.695478	6.302334	5.765265
TCGA-G3-	1.30411	0	6.402636	6.691869	5.019506	5.702274	4.760099
TCGA-UB-	1.331507	0	6.642911	6.53125	5.846285	6.200381	5.76164
TCGA-O8-	1.473973	0	6.476311	6.253341	5.754917	5.840112	5.172824
TCGA-DD-	1.512329	0	5.800904	5.936229	4.499185	5.661682	5.512967
TCGA-DD-	1.520548	0	6.127427	6.544849	4.306899	6.218041	5.724972
TCGA-DD-	1.545205	0	6.574412	6.562583	5.864995	6.102819	5.2501
TCGA-DD-	1.550685	0	6.532024	6.784625	4.801723	5.308943	3.680121
TCGA-DD-	1.572603	0	5.625349	5.70988	4.887424	5.565912	5.913018
TCGA-DD-	1.575342	0	6.525894	6.676668	5.152536	5.81652	5.567867
TCGA-RC-	1.610959	0	6.678552	6.372537	5.768507	5.06679	5.286421
TCGA-DD-	1.747945	0	6.232186	6.240525	4.955535	5.666243	5.54236
TCGA-DD-	1.764384	0	6.170819	5.915784	5.115269	5.966469	5.068223
TCGA-G3-	1.794521	0	6.114929	5.303986	5.162147	6.080831	5.260093
TCGA-DD-	1.80274	0	6.562681	6.192586	5.67674	5.967611	5.607423
TCGA-DD-	1.841096	0	5.731159	4.446074	5.089628	4.95029	5.733002
TCGA-DD-	2.090411	0	6.200492	6.346932	5.508868	5.841376	5.360289
TCGA-DD-	2.923288	0	6.30647	6.361919	5.126471	5.827149	5.360931
TCGA-DD-	2.972603	0	5.783986	5.783986	6.042425	6.265332	5.293693
TCGA-DD-	3.339726	0	6.509656	5.845534	5.278016	5.218101	5.786368
TCGA-DD-	3.372603	0	5.976364	6.219456	5.386314	5.125426	5.89737
TCGA-DD-	3.378082	0	6.555914	6.333648	5.574897	5.506936	5.223877
TCGA-DD-	3.547945	0	6.313705	5.949066	5.403605	5.990549	5.341029
TCGA-DD-	3.567123	0	6.302378	5.770478	6.164354	6.313578	5.136054
TCGA-XR-	3.668493	0	6.581917	6.286031	5.111166	5.797686	5.706305
TCGA-DD-	3.90137	0	6.064906	5.454632	5.160569	5.698051	5.917159
TCGA-DD-	3.972603	0	6.446043	6.527838	5.072991	5.826217	4.564076
TCGA-2Y-	3.978082	0	6.145884	6.369189	5.143595	5.807392	5.244258
TCGA-DD-	4.09589	0	6.157602	5.649753	5.430517	6.228321	5.798903
TCGA-DD-	4.194521	0	6.279151	6.300055	5.181519	5.443869	5.924748
TCGA-DD-	4.279452	0	6.33351	6.674142	4.465115	5.734194	5.340228
TCGA-DD-	4.293151	0	6.08878	5.818219	5.274835	5.961412	5.423522
TCGA-DD-	4.30137	0	5.775411	5.562627	4.725981	5.407061	4.968616
TCGA-2Y-	4.449315	1	6.607139	6.326132	5.312782	5.826465	5.312782
TCGA-DD-	4.994521	0	6.484849	6.657015	5.955217	6.213246	5.751938
TCGA-DD-	5.139726	0	6.302378	5.770478	5.440979	5.882598	5.249999
TCGA-DD-	5.205479	0	6.581917	6.286031	5.362682	5.986975	5.560638
TCGA-DD-	5.520548	0	6.064906	5.454632	5.36912	6.102185	5.293232
TCGA-DD-	5.556164	0	6.446043	6.527838	4.54007	5.471899	5.780589
TCGA-DD-	5.983562	0	6.145884	6.369189	4.270319	5.124882	5.212626
TCGA-DD-	6.032877	0	6.157602	5.649753	4.385964	6.012465	5.476146
TCGA-DD-	6.347945	0	6.279151	6.300055	5.671615	5.936468	5.5201
TCGA-DD-	6.884932	0	6.33351	6.674142	5.953862	5.719843	5.25252

TCGA-DD-	7.473973	0	6.08878	5.818219	5.895508	5.907665	5.686224
TCGA-DD-	7.539726	0	5.775411	5.562627	4.93828	4.871267	4.995589
TCGA-2Y-	10.06849	0	6.607139	6.326132	5.638706	5.912209	5.949438

en gene signature and HCC risk.

LBX2-AS1	MIR31HG	SEMA6A-/	LINC0127(	LINC0051(	CTBP1-AS	LINC0020(	riskScore
6.598036	5.467797	5.544474	6.289419	5.677501	5.734072	6.418455	3469.004
6.060228	5.224955	5.442542	6.796668	6.702726	5.345145	6.756209	11576.85
5.641854	5.069033	5.10958	6.084574	5.746819	4.855418	5.685242	6338.786
6.683665	5.955442	6.558604	6.832629	6.695206	6.617806	7.126636	5844.619
6.594409	6.05214	5.324645	5.974124	6.361379	5.420007	5.695042	3268.22
6.026145	5.927967	4.617974	6.110758	6.609862	5.303297	5.572877	8844.063
6.076888	5.835581	5.028664	5.508868	5.841376	5.360289	4.942906	2247.277
6.691869	5.731406	5.195691	5.126471	5.827149	5.360931	5.421472	6117.757
6.53125	5.950026	6.678042	6.042425	6.265332	5.293693	4.616783	5014.315
6.253341	5.962374	5.899229	5.278016	5.218101	5.786368	5.86327	3294.047
5.936229	5.341947	6.625627	5.386314	5.125426	5.89737	5.710692	2816.193
6.544849	5.464882	5.301474	5.574897	5.506936	5.223877	4.861826	9369.439
6.562583	6.009752	6.949441	5.403605	5.990549	5.341029	4.664388	3420.671
6.784625	5.640294	6.265881	6.164354	6.313578	5.136054	4.616017	6334.928
5.70988	5.603002	6.015328	5.111166	5.797686	5.706305	5.354859	4068.752
6.676668	5.819237	6.125712	5.160569	5.698051	5.917159	5.365515	6745.042
6.372537	6.099958	6.686027	5.072991	5.826217	4.564076	4.319113	4011.044
6.240525	5.666989	6.049617	5.143595	5.807392	5.244258	5.079854	3273.744
5.915784	5.888577	6.983047	7.079722	6.902626	6.679187	6.870045	2197.486
5.303986	5.807165	6.585578	7.071958	6.687896	6.143852	6.559177	2134.876
6.192586	5.941505	6.863585	6.865875	6.687705	6.276976	6.58115	2616.065
4.446074	5.446025	4.908997	6.45777	5.950922	5.778695	5.778695	3899.734
6.346932	5.906064	6.683716	6.545407	6.355468	5.977014	6.695799	2015.595
6.361919	5.896784	5.600079	5.534817	6.742746	4.998137	6.374121	2011.128
5.783986	5.365182	6.797107	7.134222	6.186473	5.545056	6.44083	1940.258
5.845534	5.801375	7.362478	7.192027	7.12395	6.929826	7.09118	3110.343
6.219456	5.243828	5.481299	5.833172	6.216	5.562945	6.01287	2415.08
6.333648	5.872923	6.942816	6.949081	6.849014	6.86178	6.944607	3819.881
5.949066	6.686958	6.02736	6.570485	6.232311	5.697362	6.326184	3335.689
5.770478	6.556045	6.151182	6.489374	6.423971	5.691915	6.343376	2277.738
6.286031	6.469365	6.743961	7.240866	6.591807	5.688125	6.36195	2692.243
5.454632	6.253803	5.17015	5.933915	6.489888	5.688105	5.811629	2357.625
6.527838	6.716189	6.709375	6.455638	6.708879	6.286445	6.670813	2785.035
6.369189	6.279326	5.549349	5.901263	5.901263	5.976393	5.195837	4191.313
5.649753	6.400848	6.382944	6.846748	6.551455	6.062564	6.709392	2616.655
6.300055	6.160484	5.817935	6.108363	6.533676	5.423353	6.145212	3326.649
6.674142	6.785747	6.476496	6.647522	6.33351	5.924388	6.120317	2140.098
5.818219	5.967302	6.871274	7.053274	6.807096	6.428547	6.610636	2768.597
5.562627	6.292642	4.6541	6.24646	5.188837	3.900887	5.708885	4187.793
6.326132	6.413731	6.154841	6.998426	6.717333	6.519267	6.612319	6060.214
6.657015	6.424678	7.053228	7.06214	6.911509	6.759372	6.49671	2778.923
4.843073	6.17464	6.39403	6.629375	6.717921	6.273029	6.461477	3081.643
6.223477	6.693885	4.961628	5.611267	6.636884	4.818549	6.419515	4417.112
6.084229	6.259725	6.647637	6.857118	6.31756	6.305298	6.691761	2278.181
5.853955	6.580509	7.124318	7.345441	7.037985	6.85576	6.96233	4158.267
6.163157	6.522423	6.823874	6.775507	6.867256	6.044418	7.006703	2089.273
6.05505	5.124104	5.569463	6.217989	6.164154	5.305378	5.718791	2541.634
6.371288	4.961405	5.195684	6.069223	6.637421	4.138715	5.965839	3587.501
6.497939	5.295068	6.284324	6.827556	6.778821	6.198549	6.174328	3604.523

5.996659	5.027676	5.471315	6.286909	5.68156	5.604789	6.44963	3255.055
6.253348	6.289419	6.561209	6.873425	6.914324	6.290518	6.873176	2612.596
5.192366	6.796668	6.210844	6.18511	5.670777	5.286043	5.696466	1378.95
5.409828	6.084574	6.519135	5.671736	6.275188	5.765107	5.82069	1025.888
4.003581	6.832629	5.997888	6.184702	6.161962	5.558546	5.888259	1290.155
4.626664	5.974124	5.959609	6.425407	5.817659	5.3745	5.891648	1591.784
5.051788	6.110758	4.650078	5.553093	5.11837	4.80812	5.768344	1917.699
4.937531	5.508868	5.995411	6.322123	5.571622	5.31301	5.798306	1102.923
5.290037	5.126471	6.761974	6.422642	6.222586	5.695478	6.302334	1290.048
4.356604	6.042425	5.203993	5.811329	5.412556	5.019506	5.702274	1604.142
5.314691	5.278016	6.443996	6.004046	6.107471	5.846285	6.200381	1896.762
5.452448	5.386314	6.493446	6.149022	5.614233	5.754917	5.840112	714.0084
5.146821	5.574897	5.80355	5.649279	5.765705	4.499185	5.661682	640.722
4.981891	5.403605	6.451951	6.477388	6.142523	4.306899	6.218041	1145.405
4.927618	6.164354	6.513931	6.435246	6.02836	5.864995	6.102819	1174.608
4.151155	5.111166	4.578711	5.865973	5.285687	4.801723	5.308943	967.9207
5.305361	5.160569	5.86824	5.311904	5.543378	4.887424	5.565912	893.2045
5.738411	5.072991	5.480564	5.334224	5.415983	5.152536	5.81652	1112.648
5.120227	5.143595	5.804439	5.670348	5.964112	5.768507	5.06679	1142.666
5.278984	7.079722	5.497692	5.258465	5.174581	4.955535	5.666243	1097.118
4.663483	7.071958	5.840578	6.067174	5.531158	5.115269	5.966469	1085.802
4.91875	6.865875	5.799681	5.91363	5.474022	5.162147	6.080831	1662.161
5.372784	6.45777	6.344757	6.167988	6.007444	5.67674	5.967611	1781.106
6.031217	6.545407	5.304015	5.026141	5.682672	5.089628	4.95029	1077.667
4.942906	5.534817	6.727573	6.325109	6.439029	5.508868	5.841376	765.9553
5.421472	7.134222	5.534272	5.460051	5.869356	5.126471	5.827149	1428.76
4.616783	7.192027	6.690047	6.592492	6.353042	6.042425	6.265332	1685.427
5.86327	5.833172	5.809388	4.666621	5.873987	5.278016	5.218101	571.6613
5.710692	6.949081	5.311845	5.24593	6.040544	5.386314	5.125426	1821.215
4.861826	6.570485	6.068633	5.790722	5.727705	5.574897	5.506936	743.2767
4.664388	6.489374	6.06463	6.101139	5.707395	5.403605	5.990549	1234.369
4.616017	7.240866	6.368024	6.420882	5.81552	6.164354	6.313578	1212.762
5.354859	5.933915	6.596388	6.076836	5.541564	5.111166	5.797686	459.1549
5.365515	6.455638	5.517634	5.631283	6.169318	5.160569	5.698051	1753.129
4.319113	5.901263	5.983439	6.204639	5.367462	5.072991	5.826217	426.4734
5.079854	6.846748	5.423693	5.536168	4.905653	5.143595	5.807392	1462.163
5.152843	6.108363	6.524619	6.39567	5.854028	5.430517	6.228321	1607.319
5.926663	6.647522	5.592251	5.443869	4.901023	5.181519	5.443869	1300.102
5.361304	7.053274	5.847123	5.765159	5.792738	4.465115	5.734194	1732.468
5.043949	6.24646	6.492249	5.928339	5.959888	5.274835	5.961412	737.9405
3.704974	6.998426	5.118176	5.747692	4.823302	4.725981	5.407061	1688.072
4.981225	7.06214	5.582529	5.57642	5.414546	5.312782	5.826465	1635.929
5.247247	6.629375	6.683768	6.350198	5.965974	5.955217	6.213246	1268.666
5.136091	5.611267	6.376869	6.084473	6.048538	5.440979	5.882598	1111.869
5.595534	6.857118	6.665495	6.221715	5.879259	5.362682	5.986975	488.5264
4.81489	7.345441	6.280339	5.80495	5.698731	5.36912	6.102185	616.739
5.719452	6.775507	6.096707	5.105504	5.866157	4.54007	5.471899	620.5473
4.585792	6.217989	4.451743	5.09032	4.270319	4.270319	5.124882	818.23
5.596366	6.069223	6.487243	5.37795	5.665039	4.385964	6.012465	717.9866
5.139492	6.827556	6.545958	6.068377	6.170857	5.671615	5.936468	1091.601
5.080576	6.286909	6.339379	6.386888	5.861842	5.953862	5.719843	1194.365

5.211922	7.345441	6.591426	6.415611	6.067693	5.895508	5.907665	1311.994
4.395039	7.345441	5.045514	5.726163	5.927216	4.93828	4.871267	1519.747
5.6945	7.345441	5.807348	5.253436	5.883866	5.638706	5.912209	1916.005

risk
high
high
high
high
high
high
high
high
high
high
high
high
high
high
high
high
high
high
high
high
high
high
high
high
high
high
high
high
high
high
high
high
high
high
high
high
high
high
high
high
high
high
high
high
high
high
high
high
high

high
high
low
low
low
low
low
low
low
low
low
low
low
low
low
low
low
low
low
low
low
low
low
low
low
low
low
low
low
low
low
low
low
low
low
low
low
low
low
low
low
low
low
low
low
low
low
low
low
low
low

low
low
low
