# Supplementary material for: Immune-Related lncRNAs with WGCNA Identified the Function of SNHG10 in HBV-Related Hepatocellular Carcinoma
Source: J Oncol. 2022 Jul 6;2022:9332844. doi: 10.1155/2022/9332844 (PMC9279027; doi:10.1155/2022/9332844)
Supplement: Supplementary Materials — Supplementary table 1: immune‐related gene expressions in HBV-related hepatocellular carcinoma from TCGA database for the WGCNA analysis. Supplementary table 2: the clinical characteristics of these eligible patients. Supplementary table 3: list of immune-related genes in the co-expression modules. Supplementary table 4: pathway analysis mapped the identification in the red co-expression module. Supplementary table 5: the co-expression analysis between immune-related genes in the red co-expression module and lncRNAs. Supplementary table 6: 33 immune-related lncRNAs were significant related to the overall survival. Supplementary table 7: lasso regression was constructed examining the relationship between gene signature and HCC risk. Supplementary table 8: quantification of the abundance of immune cell infiltration in tumor microenvironment by CIBERSORT web portal with the LM22 signature. [file 9332844.f1.zip › Supplementary table 8.pdf]

Supplementary table 8: Quantification of the abundance of immune cell infiltration in tum

Mixture	B cells naiv	B cells mer	Plasma cell	T cells CD8	T cells CD4	T cells CD4	T cells CD4
TCGA-2Y-	0.017988	0	0.026629	0.404684	0	0.06642	0
TCGA-2Y-	0	0	0.086434	0.417925	0	0	0.113982
TCGA-2Y-	0	0	0	0.093987	0	0.186246	0
TCGA-5C-	0.010982	0	0.176649	0.12599	0	0.06221	0.013186
TCGA-BC-	0.027112	0	0.002743	0.038002	0	0.094087	0
TCGA-BW	0.066693	0	0.001766	0.148797	0	0.097982	0
TCGA-CC-	0.02605	0	0.083784	0.122792	0	0.159274	0
TCGA-DD-	0.007324	0	0.020852	0.064518	0.001918	0.034791	0
TCGA-DD-	0.035997	0	0.010457	0.073018	0	0.254852	0
TCGA-DD-	0.091447	0	0.076621	0.019681	0	0.207545	0
TCGA-DD-	0.028953	0	0.035901	0.056896	0	0.312826	0
TCGA-DD-	0.023674	0	0	0.044444	0	0.306814	0
TCGA-DD-	0.002709	0	0.006256	0	0	0.063246	0.005571
TCGA-DD-	0	0.001352	0	0.03753	0	0.174838	0
TCGA-DD-	0.108435	0	0	0	0	0.194345	0
TCGA-DD-	0.039443	0	0.038115	0.104215	0	0.240133	0
TCGA-DD-	0.02415	0	0.035674	0.104191	0	0.127107	0
TCGA-DD-	0.156144	0	0.033338	0	0	0.135018	0
TCGA-DD-	0.010952	0	0.002601	0	0	0.335334	0
TCGA-DD-	0.011136	0	0.148191	0.355288	0	0	0.048149
TCGA-DD-	0.051478	0	0.049494	0.351374	0	0.021978	0.032796
TCGA-DD-	0	0	0	0.041955	0	0.305568	0
TCGA-DD-	0.097482	0	0.098028	0.094585	0	0.193494	0
TCGA-DD-	0.026652	0	0.009629	0.050756	0	0.216942	0
TCGA-DD-	0.040546	0	0.001828	0	0	0.189156	0
TCGA-DD-	0.072551	0	0.006952	0.070002	0	0.312341	0
TCGA-DD-	0.015978	0	0.02164	0.060989	0	0.356138	0
TCGA-DD-	0.010484	0	0	0.055008	0	0.270813	0
TCGA-DD-	0.140949	0	0.014586	0.102854	0	0.091742	0
TCGA-DD-	0.041434	0	0	0.088315	0	0.102773	0
TCGA-DD-	0.029329	0	0.038389	0.23962	0	0.171893	0
TCGA-DD-	0	0.001265	0.011131	0.130731	0	0.285769	0
TCGA-DD-	0.043923	0	0	0.068111	0	0.334944	0
TCGA-DD-	0.050042	0	0	0.013945	0	0.113568	0.017773
TCGA-DD-	0.079248	0	0	0.14902	0	0.262153	0
TCGA-DD-	0.061588	0	0.012207	0.078357	0	0.150182	0.014806
TCGA-DD-	0.093109	0.006	0	0.17986	0	0.359099	0
TCGA-DD-	0.052789	0	0	0.03756	0	0.252809	0.005961
TCGA-DD-	0.061567	0	0.014581	0.025879	0	0.325832	0
TCGA-DD-	0.004028	0	0.009886	0	0	0.157252	0
TCGA-DD-	0.01538	0	0	0.087264	0	0.308425	0
TCGA-DD-	0.059768	0	0.004224	0.110491	0	0.22088	0
TCGA-DD-	0.077149	0	0.006991	0.175374	0	0.340796	0
TCGA-DD-	0.029277	0	0.041278	0.248794	0	0.057156	0.020198
TCGA-DD-	0.040355	0	0.031928	0.058657	0	0.131529	0
TCGA-DD-	0.056864	0	0.022008	0.251047	0	0	0.030154
TCGA-DD-	0.054137	0	0.243019	0.299235	0	0	0.069595
TCGA-DD-	0.030849	0	0.045226	0.235532	0	0.108198	0
TCGA-DD-	0.037708	0	0.015646	0.192423	0	0.408544	0
TCGA-DD-	0.115808	0	0.038653	0.083731	0	0.261485	0
TCGA-DD-	0.085043	0	0	0.087194	0	0.181409	0
TCGA-DD-	0.014497	0	0.017417	0.105272	0	0.207825	0

TCGA-DD-	0	0.003807	0.106455	0.340514	0	0	0.107566
TCGA-DD-	0.009546	0	0.058919	0.158604	0	0.076583	0.015993
TCGA-DD-	0.068028	0	0.001627	0.05352	0	0.303462	0.014145
TCGA-DD-	0.011677	0	0	0.229775	0	0.037251	0
TCGA-DD-	0.006419	0	0.01705	0.104119	0	0.398633	0
TCGA-DD-	0.04074	0	0.004851	0.07473	0	0.13634	0
TCGA-DD-	0.036588	0	0.001057	0.071756	0	0.232098	0
TCGA-DD-	0.039944	0	0	0.055756	0	0.328312	0
TCGA-DD-	0.006056	0	0	0.074427	0	0.153804	0
TCGA-DD-	0.056203	0	0	0.058791	0	0.26886	0
TCGA-DD-	0.05665	0	0.042359	0.178099	0	0.139373	0
TCGA-DD-	0.022103	0	0.006717	0.05938	0	0.081428	0.003572
TCGA-DD-	0.013889	0	0.027415	0.151264	0	0.17938	0
TCGA-DD-	0.002677	0	0.040393	0.173576	0	0.226026	0.0022
TCGA-DD-	0.038153	0	0	0.131681	0	0.0944	0
TCGA-DD-	0.036928	0	0.014103	0.239391	0	0.01976	0.071021
TCGA-DD-	0.014999	0	0	0.126927	0	0.156971	0
TCGA-DD-	0.066707	0	0.006408	0.017635	0	0.203157	0
TCGA-DD-	0.065159	0	0.00693	0	0	0.16333	0.020929
TCGA-DD-	0	0	0	0.128538	0	0.149474	0
TCGA-DD-	0.043356	0	0.011599	0.168335	0	0.142831	0
TCGA-DD-	0.085151	0	0.059783	0.163473	0	0.033001	0.00306
TCGA-DD-	0.074795	0	0.084697	0.18521	0	0.140277	0.004198
TCGA-DD-	0.023405	0	0	0.086679	0	0.437405	0.057446
TCGA-DD-	0.003562	0	0	0.154474	0	0.293887	0
TCGA-DD-	0	0	0	0.011863	0	0.123943	0
TCGA-DD-	0.027125	0	0.029578	0.065631	0	0.192143	0.029554
TCGA-DD-	0.015654	0	0	0.02598	0	0.466137	0
TCGA-DD-	0.057415	0	0	0.103628	0	0.247243	0
TCGA-DD-	0.080378	0	0	0.03337	0	0.403823	0
TCGA-DD-	0.033296	0	0	0.029215	0	0.253199	0
TCGA-G3-	0.103415	0	0	0	0	0.222828	0
TCGA-G3-	0	0	0	0.176136	0	0.112684	0
TCGA-G3-	0.018427	0	0	0.070562	0	0.292823	0
TCGA-G3-	0.001626	0	0.002353	0.062847	0	0.188684	0
TCGA-G3-	0.061914	0	0	0.123975	0	0.234253	0
TCGA-G3-	0.050271	0	0	0.175768	0	0.178729	0
TCGA-G3-	0.023072	0	0.079027	0.148596	0	0.189296	0
TCGA-G3-	0	0	0.025529	0.161216	0	0.206784	0
TCGA-G3-	0.102533	0	0	0	0	0.144747	0
TCGA-G3-	0.005329	0	0.010758	0	0	0.330942	0
TCGA-G3-	0	0.003684	0	0.02604	0	0.068948	0
TCGA-K7-	0.000853	0	0.022134	0.108038	0	0.120923	0
TCGA-O8-	0.018733	0	0.006598	0.185575	0	0.143233	0.008066
TCGA-QA-	0.056891	0	0	0.056499	0	0.220383	0
TCGA-RC-	0.047322	0	0.075139	0.086284	0	0.122756	0.015243
TCGA-RC-	0.044543	0	0	0.073911	0	0.183853	0
TCGA-RC-	0.055778	0	0	0.134979	0	0.162065	0
TCGA-UB-	0.009568	0	0	0	0.007125	0.241298	0
TCGA-UB-	0.047933	0	0.028895	0.166321	0	0.12922	0
TCGA-UB-	0.047111	0	0.049556	0.287501	0	0.077261	0
TCGA-XR-	0.022204	0	0	0.055652	0	0.474048	0
TCGA-XR-	0.056749	0	0.011434	0.245908	0	0.120092	0.038054
TCGA-ZP-	0.00371	0.001155	0.090386	0.135906	0	0.162967	0.004046

ıor microenvironment by CIBERSORT web portal with the LM22 signature.

T cells folli	T cells regu	T cells gam	NK cells re	NK cells ac	Monocytes	Macrophag	Macrophag
0.062933	0.122847	0	0	0.037746	0.02525	0.015053	0.069379
0.041661	0.047794	0	0	0.027573	0.012743	0.04602	0.068241
0	0.103384	0.000873	0	0.083448	0	0	0.060848
0.006855	0.068391	0	0	0.031529	0	0.147342	0.157367
0.036516	0.057375	0	0	0.033084	0.026805	0.133047	0.105926
0	0.048858	0.009274	0	0.077297	0.040853	0.083598	0.166226
0.069755	0.086947	0	0.000897	0.031092	0	0.143392	0.092674
0	0.106833	0.012714	0	0.095674	0.008031	0.165286	0.073843
0	0.011401	0	0	0.061863	0	0.256051	0.040222
0	0.005369	0.042871	0	0.057687	0.002797	0.078044	0.170229
0.00418	0.003807	0	0	0.071109	0.00893	0.118928	0.102427
0	0.054234	0	0	0.067465	0.019156	0.114982	0.058612
0.013839	0.06518	0	0.048125	0	0.01174	0.352348	0.02843
0	0.072603	0	0	0.063923	0.034852	0.265343	0.078326
0	0.048793	0	0	0.088812	0.037478	0.116304	0.085451
0.000169	0.077048	0	0	0.030299	0.009623	0.130151	0.064028
0.00462	0.005936	2.04E-05	0	0.112043	0.018764	0.119169	0.0875
0	0.078492	0	0.057788	0.082828	0.096553	0.123241	0.017999
0	0.040001	0	0.049775	0.106225	0.031787	0.167187	0.027787
0.027047	0.055172	0	0	0.040788	0.02485	0.056392	0.079121
0.022321	0.088935	0.020859	0	0.065165	0.014425	0.058018	0.074732
0	0.029537	0.035608	0	0.050662	0.075518	0.049573	0.07677
0.006595	0.057498	0	0	0.069291	0.034308	0.048814	0.113189
0	0.048616	0.049134	0	0.016292	0	0.218586	0.021452
0.002256	0.106961	0	0	0.124467	0.012557	0.193522	0.028523
0.024712	0.050743	0	0	0.047952	0.011855	0.033969	0.108742
0	0.002103	0	0	0.051972	0.033835	0.058758	0.159175
0.002769	0.021878	0.042071	0	0.058651	0.005159	0.0394	0.089427
0.104695	0.003789	0	0.022924	0.057574	0.007706	0.036801	0.225044
0.048686	0.047843	0	0	0.089729	0.024987	0.128578	0.109087
0	0.028564	0	0	0.069154	0.031737	0.050141	0.089002
0	0.052036	0	0	0.033425	0.011335	0.066544	0.133844
0	0.038108	0.014006	0	0.043035	0.00847	0.002323	0.040751
0.002134	0	0.049986	0	0.066299	0.07724	0.100098	0.147647
0.001483	0.024614	0.072524	0	0.075983	0	0.055038	0.072456
0	0.072604	0	0	0.04269	0.01835	0.178939	0.103217
0	0.061506	0	0	0.035663	0.005094	0.055014	0.047226
0	0.047254	0	0.011705	0.013241	0.004971	0.281535	0.106388
0.015517	0.01006	0	0	0.001672	0.011087	0.173402	0.231219
0.002421	0.037189	0	0.004838	0.080835	0.054927	0.022382	0.020728
0	0.121859	0	0	0.077132	0.091634	0.129633	0.040072
0.030046	0.00379	0.057843	0	0.103135	0	0.036083	0.083378
0.004675	0.011603	0.064247	0	0.042626	0	0.033533	0.079148
0.032286	0.111287	0	0	0.053231	0.010926	0.201	0.05855
0.046304	0.065212	0.004147	0	0.075941	0	0.188961	0.141783
0.111351	0.013125	0	0	0.033867	0.0092	0.079402	0.095605
0.046499	0.052706	0	0	0.043103	0	0.034505	0.077476
0.019161	0	0	0.001265	0.107017	0.033742	0.075986	0.07372
0.002692	0.008398	0.0219	0	0	0.009147	0.078023	0.090831
0	0.059208	0	0	0.033344	0.02006	0.058508	0.073924
0	0	0	0.056631	0.080846	0.013554	0.081913	0.137841
0	0.097277	0	0	0.075929	0.014707	0.09792	0.071937

0.028584	0.084132	0	0.002389	0.033173	0.025575	0.11001	0.062515
0.070134	0.159629	0	0	0.040026	0.019834	0.204715	0.054258
0	0.065208	0	0	0.050752	0.015354	0.071381	0.14542
0.041622	0.10128	0	0.033604	0.107972	0.020516	0.102454	0.086366
0	0.007704	0.056516	0	0.051218	0	0.109795	0.045885
0.000614	0.063734	0	0	0.052502	0	0.177917	0.044403
0.030822	0.011314	0	0	0.066293	0	0.215442	0.087336
0	0.042913	0	0	0.043939	0.012334	0	0.126984
0	0.077523	0	0	0.073661	0	0.387437	0.109426
0	0.093613	0.011742	0	0.075672	0.009534	0.02573	0.069768
0.04177	0.007975	0.064908	0	0.036193	0	0.012005	0.214528
0.000367	0	0.022325	0	0.098881	0.037461	0.063818	0.058574
0.016234	0.024408	0.055504	0	0.099516	0	0.036104	0.150134
0	0	0.063547	0	0.011376	0	0.002703	0.105136
0.005677	0.070685	0	0	0.074	0.023582	0.151482	0.081519
0.084818	0.101489	0.03993	0	0.019951	0	0.113524	0.100617
0	0.029206	0	0.048873	0.153247	0	0.097922	0.040951
0.034128	0.064858	0.011669	0.026814	0	0	0.246018	0.067258
0	0.015295	0.055846	0.085356	0	0.017872	0.179663	0.126081
0.001166	0.069483	0.009011	0	0.083728	0	0.146	0.073863
0.030354	0.074733	0	0	0.05575	0.007708	0.192614	0.08413
0.052936	0.004904	0	0.008193	0.055924	0.134039	0	0.146013
0.063662	0.056382	0	0	0.013159	0.020565	0.169527	0.063691
0	0.030363	0	0	0	0.019645	0.000326	0.048823
0	0.021292	0	0	0.068695	0.027935	0.080914	0.057904
0	0	0.024231	0	0.039876	0	0.53203	0.030056
0	0.062634	0	0	0.034802	0.009671	0.155432	0.136052
0	0.067046	0	0	0.031417	0.010901	0.046392	0.02837
0	0.038701	0.024506	0	0.053054	0.029033	0	0.137508
0	0.025456	0	0	0.05926	0.01271	0.018209	0.069146
0	0.061937	0	0	0.017322	0.020141	0.352718	0.094669
0	0.000274	0	0.048123	0.017203	0.076262	0.000691	0.125674
0	0.096057	0	0	0.101511	0.005321	0.096243	0.062826
0.041348	0.038671	0.003112	0	0.027863	0.003578	0.039964	0.141628
0	0.094319	0	0.060582	0	0.034931	0.107438	0.054483
0	0.086798	0.04475	0	0.042926	0.002189	0	0.090277
0.008111	0.013098	0	0	0.070159	0.020159	0.087621	0.16307
0.079587	0.040152	0.014419	0	0.009976	0.010203	0	0.128499
0.005245	0.035518	0	0	0.131478	0.02945	0.013588	0.131349
0	0.052932	0.020203	0	0.047417	0.019209	0.105776	0.060748
0	0.021978	0.10415	0	0.057321	0.044925	0.000572	0.093002
0.03001	0.055869	0	0	0.010761	0	0.628026	0.040386
0.033569	0.100711	0.028945	0	0.079229	0.02304	0.059711	0.161503
0	0.085636	0	0	0.025964	0.015814	0.13486	0.058163
0.072281	0.093333	0.031178	0	0.125915	0	0	0.024386
0.038134	0.071309	0.014382	0.006753	0.016659	0.008559	0.108452	0.163499
0.040691	0.026876	0	0	0.048161	0	0.133931	0.129948
0.058437	0.12439	0	0	0.108327	0.002905	0.140048	0.082874
0.017885	0	0	0.040809	0.047732	0.09461	0.033798	0.080025
0.03535	0.102415	0.004509	0	0.078334	0	0.194913	0.088626
0.008359	0.098793	0	0.005256	0.042837	0.032978	0.019572	0.164538
0	0.014042	0	0	0.046473	0.004931	0.020429	0.059724
0.077142	0.044239	0.015626	0	0	0.010623	0.082918	0.081909
0.019238	0.068142	0	0	0.017933	0.010775	0.043361	0.146762

Macrophag	Dendritic c	Dendritic c	Mast cells	Mast cells	Eosinophils	Neutrophil	P-value
0.14138	0	0	0.009691	0	0	0	0.09
0.111055	0.004363	0	0.022208	0	0	0	0.17
0.213573	0.19489	0	0.062751	0	0	0	0.5
0.110637	0.021562	0	0.065004	0	0	0.002296	0
0.257716	0.147552	0	0.040033	0	0	0	0.41
0.124969	0.06425	0	0.069438	0	0	0	0.69
0.067991	0.064109	0	0.051243	0	0	0	0.67
0.258384	0.012264	0	0.137569	0	0	0	0.11
0.174642	0.045576	0	0.035921	0	0	0	0.09
0.118522	0.019549	0	0.108564	0	0	0.001074	0.1
0.171468	0.01179	0	0.068817	0	0	0.003967	0.25
0.207936	0.021604	0	0.075312	0	0	0.005768	0.5
0.277319	0.052738	0	0.071173	0	0.001326	0	0.01
0.181939	0	0	0.040715	0	0	0.048578	0.26
0.303237	0.002437	0	0	0	0	0.014708	0.16
0.215649	0.009465	0	0.031295	0	0	0.010367	0.11
0.102412	0.008357	0	0.244277	0	0	0.00578	0.34
0.062259	0.015489	0	0.119593	0	0	0.021259	0.31
0.09695	0	0	0.131401	0	0	0	0.31
0.113077	0.025161	0	0.015629	0	0	0	0.1
0.111339	0.03368	0	0	0	0	0.003406	0
0.257771	0	0.046005	0.031033	0	0	0	0.17
0.119808	0.018581	0	0.048328	0	0	0	0.41
0.282958	0.032972	0	0.026012	0	0	0	0.09
0.253904	0	0	0.029772	0	0	0.016509	0.5
0.185298	0.047145	0	0.026134	0	0.001603	0	0.73
0.111923	0.031	0	0.096488	0	0	0	0.36
0.177818	0.041777	0	0.183649	0	0	0.001094	0.35
0.179858	0	0	0	0	0	0.011476	0.33
0.168298	0.038447	0	0.092158	0	0	0.019664	0.72
0.180208	0.029633	0	0.039059	0	0	0.003271	0.31
0.088399	0.130034	0	0.055488	0	0	0	0.31
0.244289	0.09157	0	0.070472	0	0	0	0.94
0.156484	0.030363	0	0.165238	0	0	0.009184	0.19
0.177593	0.029888	0	0	0	0	0	0.72
0.183331	0.040089	0	0.03588	0	0	0.00776	0.1
0.084434	0.036034	0	0.033366	0	0	0.003595	0.18
0.140268	0.01658	0	0.028939	0	0	0	0.09
0.026173	0.021439	0	0.081015	0	0	0.000556	0.09
0.406928	0	0.016875	0.176131	0	0.002921	0.00266	0.12
0.106285	0	0	0.022316	0	0	0	0.52
0.198442	0.000147	0	0.088224	0	0	0.003548	0.82
0.11518	0.016407	0	0.032269	0	0	0	0.53
0.082904	0.03208	0	0.021032	0	0	0	0.1
0.11494	0.087326	0	0.012915	0	0	0	0.22
0.244703	0.008997	0	0.043675	0	0	0	0.46
0.055431	0	0	0.024294	0	0	0	0.39
0.135526	0.00671	0	0.127068	0	0	0	0.37
0.099819	0.018225	0	0.016644	0	0	0	0.08
0.142263	0.074594	0	0.038422	0	0	0	0.16
0.18129	0.009078	0	0.068984	0	0	0.016219	0.43
0.218932	0.013669	0	0.064618	0	0	0	0.42

0.077137	0.004562	0	0.013583	0	0	0	0
0.096255	0.012366	0	0.023137	0	0	0	0.09
0.082057	0.061676	0	0.050964	0	0	0.016407	0.5
0.120822	0.016972	0	0.089689	0	0	0	0.55
0.143429	0.029735	0	0.029496	0	0	0	0.27
0.251717	0.106852	0	0.04179	0	0	0.003809	0.2
0.148571	0.015154	0	0.08357	0	0	0	0.17
0.245358	0.073474	0	0.030985	0	0	0	0.67
0.07962	0.007901	0	0.030145	0	0	0	0.07
0.14927	0.095493	0	0.085325	0	0	0	0.72
0.143758	0.029724	0	0.032657	0	0	0	0.01
0.541711	0	0	0	0.003663	0	0	0.36
0.147895	0.022179	0	0.073657	0	0	0.002421	0.01
0.2266	0.135974	0	0.009792	0	0	0	0.45
0.239397	0.0082	0	0.081222	0	0	0	0.35
0.114744	0.016708	0	0.027015	0	0	0	0.1
0.217136	0.00086	0	0.112908	0	0	0	0.41
0.23836	0	0	0.016987	0	0	0	0.09
0.139356	0.023555	0	0.100626	0	0	0	0.19
0.257155	0.016078	0	0.065505	0	0	0	0.18
0.107833	0.028614	0	0.052143	0	0	0	0.07
0.17328	0	0	0.076558	0	0	0.003686	0.45
0.055112	0.031083	0	0.037643	0	0	0	0.01
0.166775	0.100386	0	0.012293	0	0	0.016455	0.09
0.086177	0.14468	0	0.060482	0	0	0	0.53
0.199144	0.002958	0	0.035899	0	0	0	0.01
0.13625	0.067502	0	0.053056	0	0	0.00057	0.01
0.163106	0.114365	0	0.030633	0	0	0	0.38
0.242333	0.010778	0	0.055801	0	0	0	0.23
0.132546	0.119161	0	0.045939	0	0	0	0.84
0.085532	0.004844	0	0.047125	0	0	0	0.01
0.181377	0.002949	0	0.151363	0	0	0.069841	0.66
0.193353	0.084039	0	0.04033	0	0	0.0315	0.01
0.23707	0.009739	0	0.074143	0	0	0.001074	0.18
0.245443	0.044653	0	0.091614	0	0.000754	0.010272	0.19
0.2236	0.017819	0	0.071499	0	0	0	0.66
0.119833	0	0	0.106272	0	0.006908	0	0.19
0.225182	0.026104	0	0.025887	0	0	0	0.22
0.132352	0.00933	0	0.118162	0	0	0	0.84
0.227821	0.156005	0	0.027537	0	0.035072	0	0.34
0.165145	0	0	0.162464	0	0	0.003414	0.66
0.12112	0.002347	0	0.012517	0	0	0.000293	0.01
0.200091	0.012008	0	0.047472	0	0	0.001774	0.31
0.219462	0.044695	0	0.053201	0	0	0	0.19
0.157071	0.022394	0.003551	0.069	0	0	0.067117	0.7
0.126616	0.059734	0	0.03916	0	0	0	0.69
0.221913	0.035357	0	0.060816	0	0	0	0.11
0.053365	0.010836	0	0.065996	0	0	0	0.46
0.233422	0.005741	0	0.170435	0	0.00877	0.008782	0.55
0.08011	0	0	0.038865	0	0	0.004508	0.04
0.076438	0.040783	0	0.049018	0	0	0	0
0.217137	0.047876	0	0.037484	0	0	0	0.94
0.215306	0	0	0	0	0	0	0.68
0.132022	0.130242	0	0.032373	0	0	0.000984	0.11

Correlatior	RMSE
0.112244	1.077594
0.061887	1.082619
0.007292	1.090028
0.446785	0.893801
0.018117	1.095433
-0.01079	1.112467
-0.00833	1.088867
0.080917	1.067102
0.108697	1.061401
0.09675	1.057175
0.045289	1.077205
0.006506	1.091668
0.184849	1.051872
0.043842	1.096809
0.068761	1.074691
0.07936	1.060135
0.030208	1.086767
0.036658	1.064799
0.036624	1.087293
0.098294	1.052495
0.350171	0.946354
0.062729	1.073571
0.020169	1.076434
0.109617	1.062808
0.007086	1.106995
-0.01727	1.10578
0.02832	1.095123
0.028534	1.082483
0.032423	1.119704
-0.01593	1.100949
0.037161	1.084747
0.036967	1.087809
-0.03289	1.10392
0.055755	1.077491
-0.01639	1.12257
0.088795	1.059609
0.059611	1.087953
0.105901	1.067523
0.108678	1.082748
0.075481	1.068082
0.004789	1.103904
-0.02499	1.115517
0.003687	1.119233
0.087303	1.064357
0.049939	1.085553
0.01131	1.089403
0.022168	1.078458
0.025954	1.086263
0.121656	1.066638
0.068471	1.055539
0.014765	1.099617
0.014964	1.089344

0.253404	0.987074
0.100779	1.054065
0.006434	1.099656
0.001486	1.110301
0.042322	1.094906
0.05359	1.077753
0.065125	1.074677
-0.00636	1.105373
0.134103	1.075316
-0.01497	1.095214
0.188972	1.040715
0.028454	1.125235
0.183664	1.033568
0.012681	1.096835
0.028748	1.086531
0.086208	1.065404
0.01859	1.107331
0.11169	1.06012
0.055672	1.073277
0.060789	1.079605
0.136228	1.04146
0.013114	1.086972
0.197032	1.00555
0.112282	1.053877
0.004367	1.096993
0.189314	1.091357
0.191498	1.014722
0.023592	1.093338
0.048245	1.084817
-0.02674	1.104667
0.180335	1.038854
-0.00215	1.090788
0.20223	1.012886
0.060981	1.079331
0.054649	1.065772
-0.00407	1.101316
0.054414	1.089406
0.050899	1.075277
-0.02718	1.129379
0.030367	1.076556
-0.00264	1.100904
0.19362	1.110746
0.037251	1.097021
0.055054	1.072338
-0.01222	1.109363
-0.01024	1.099553
0.08097	1.067579
0.010393	1.105053
0.001491	1.086738
0.161387	1.03419
0.271381	0.988286
-0.03285	1.122101
-0.00874	1.106849
0.081072	1.058668
